# Supplementary material for: Interneuron-specific signaling evokes distinctive somatostatin-mediated responses in adult cortical astrocytes
Source: Nat Commun. 2018 Jan 8;9:82. doi: 10.1038/s41467-017-02642-6 (PMC5758790; doi:10.1038/s41467-017-02642-6)
Supplement: Supplementary file 1 — Supplementary Information [file 41467_2017_2642_MOESM1_ESM.pdf]

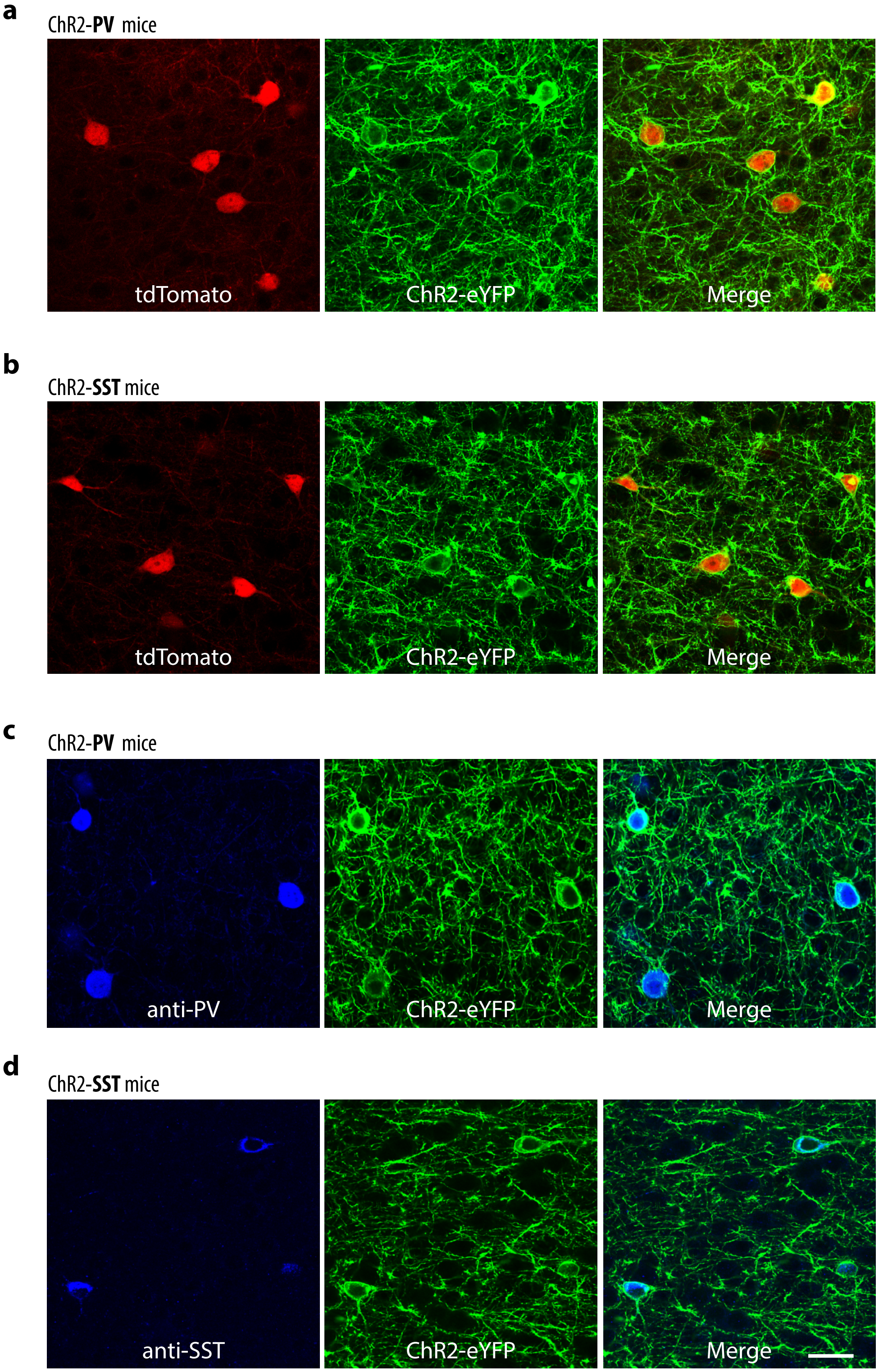

Supplementary Figure 1. ChR2 expression in ChR2-PV and ChR2-SST mice. (a) Confocal images of a SSCx slice from a PV-Cre/tdTomato mouse injected with AAV2/1-ChR2-eYFP showing tdTomato (red) and ChR2-eYFP (green) expressing cells. Merge is also shown. Quantitative analysis revealed that 83.3 ± 1.9% of tdTomato positive cells are ChR2 positive (2082 tdTomato cells analyzed). (**b**) Same as in (a), but for SST-Cre/tdTomato mice injected with AAV2/1-ChR2-eYFP. 83.1 ± 1.8% of tdTomato positive cells are ChR2 positive (1798 tdTomato cells analyzed). (**c**) Confocal images of a SSCx slice from a PV-Cre mouse injected with AAV2/1-ChR2-eYFP showing blue PV (anti-PV staining; see Methods) and green ChR2-eYFP expressing cells. Merge is also shown. 80.6 ± 2.4% of PV positive cells are ChR2 positive (1288 PV cells analyzed). **(d)** Same as in (c), but for SST-Cre mice injected with AAV2/1-ChR2-eYFP and anti-SST staining. 96.2 ± 2.1% of SST positive cells are ChR2 positive (536 SST cells analyzed). Scale bar, 25 µm.

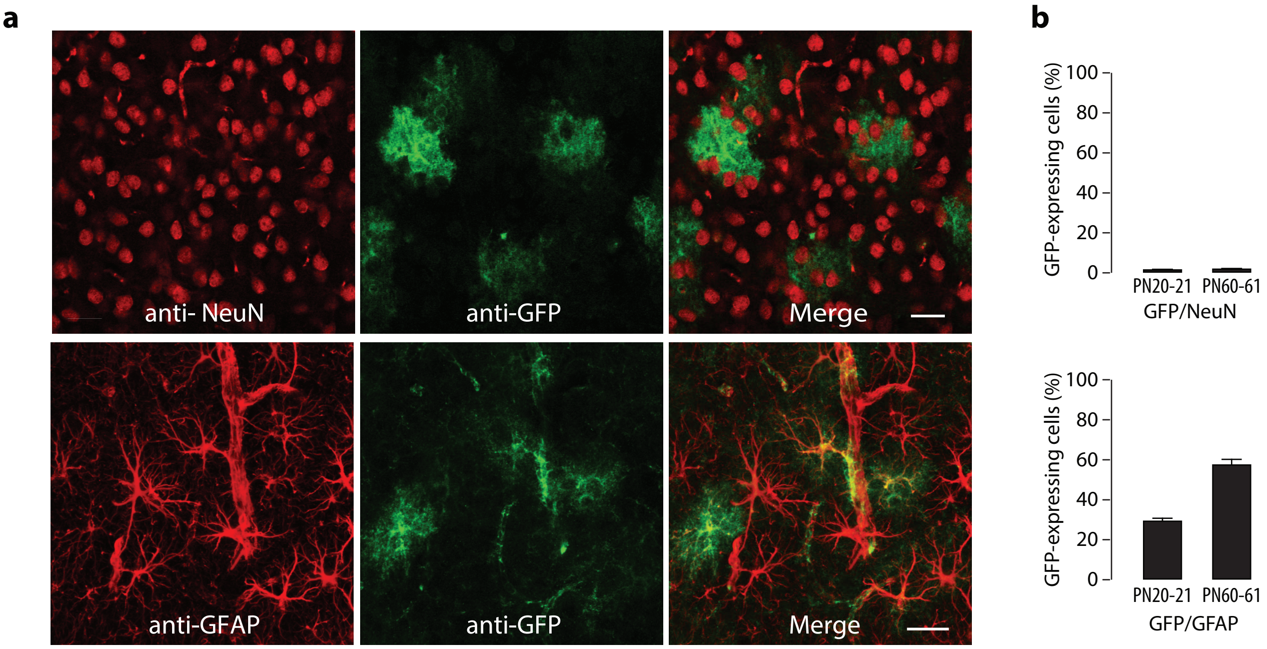

Supplementary Figure 2. GCaMP6f expression in ChR2-SST mice. (a) Confocal representative images of the SSCx from a SST-Cre-mouse injected with both AAV2/1.EF1.-dflox.hChR2(H134R)-mCherry. WPRE.hGH and AAV2/5.GfaABC.cyto.GCaMP6f showing red fluorescent neurons (anti-NeuN staining, top left panel) or astrocytes (anti-GFAP staining, bottom left panel) and green GCaMP6f-expressing cells (anti-GFP staining; see Methods). Merge images are also shown (right panels). Scale bars, 25 µm. **(b)** Summary of the mean percentage of GCaMP6f-expressing cells in SSCx from mice at different ages. PN 20-21 mice: 168 GFP-positive cells out of 18340 NeuN positive cells (67 z-stacks, 7 slices, 2 mice) and 555 GFP-positive cells out of 1829 GFAP positive cells (76 z-stacks, 8 slices, 2 mice). PN 60-61 mice: 300 GFP-positive cells out of 17180 NeuN positive cells (96 z-stacks, 11 slices, 2 mice) and 1255 GFP positive cells out 2180 GFAP positive cells (98 z-stacks, 10 slices, 2 mice). Data are represented as mean ± SEM.

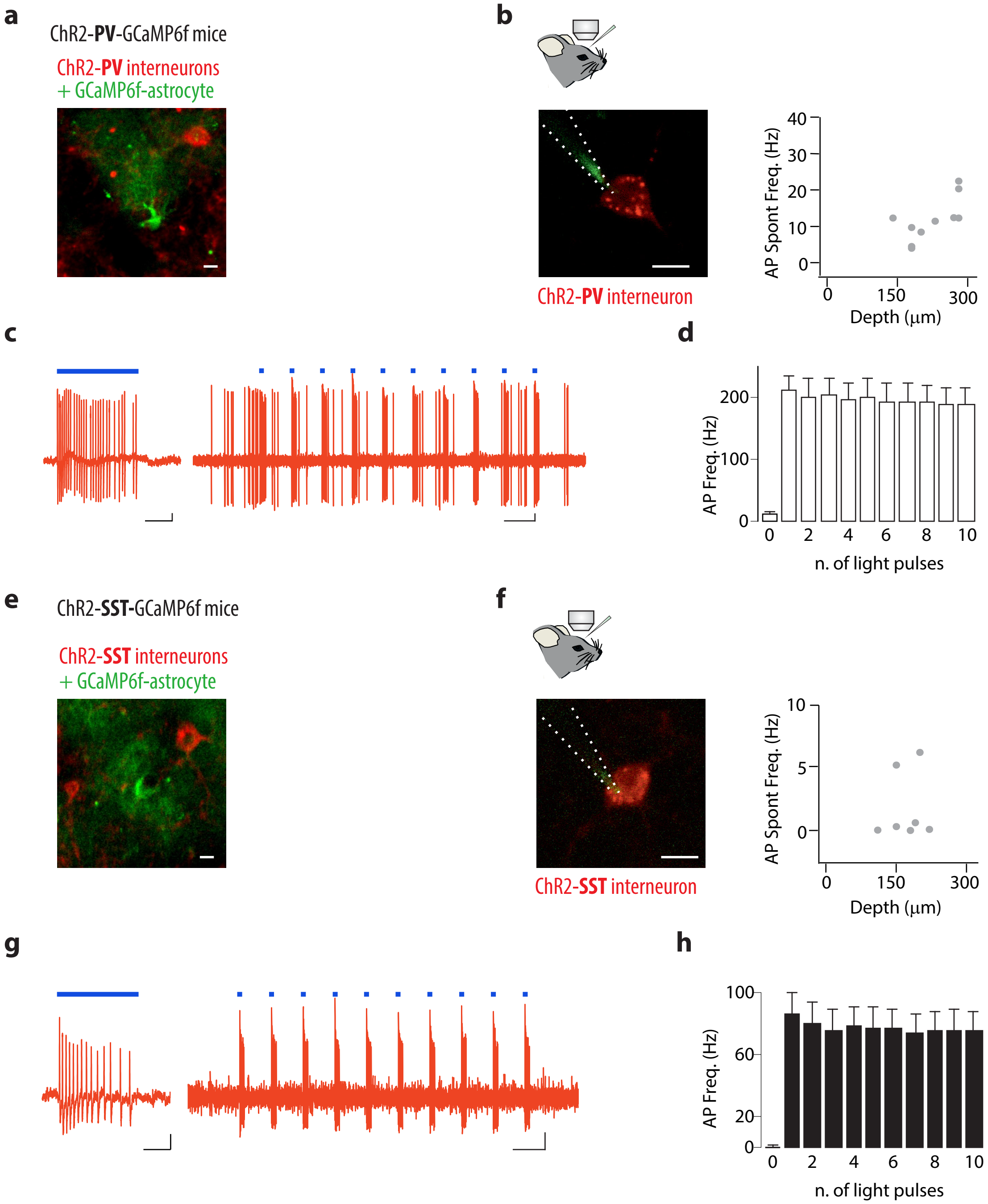

Supplementary Figure 3. ***In vivo*** **imaging of ChR2-PV- or ChR2-SST-GCaMP6f mice and juxtasomal recordings upon light stimulation of ChR2-PV and ChR2-SST expressing interneurons.** (**a**). *In vivo* two-photon image from a ChR2-PV-GCaMP6f mouse showing a GCaMP6f astrocyte and a ChR2-PV-mCherry-expressing interneuron in SSCx layer 2. Scale bar, 10 μm. (**b**). Left, schematic of the electrophysiological *in vivo* experiment and two-photon image of a SSCx layer 2 PV-ChR2-mCherry-expressing cell with indication of the patch pipette for the juxtaposed recording. Right, quantification of the spontaneous AP firing frequency from the different PV interneurons at different depth below tissue surface. Scale bar, 10 μm (**c**) Representative juxtaposed recordings of AP firing from a ChR2-PV interneuron evoked by a single and by 10 subsequent light pulses (150 ms duration, 1 Hz). Scale bars, 50 ms, 1s, 0.2 mV. (**d**) Summary of AP frequency evoked by 10 subsequent light pulses as in (c) from ChR2-mCherry PV interneurons (n = 10 cells, 2 mice). The mean frequency rate was 11.77 ± 1.88 Hz at rest (spontaneous activity), about 200 Hz during the pulse and 40.8 ± 5.75 Hz during the overall stimulation period. Data are represented as mean ± SEM. (**e**) same as in (a) but for a ChR2-SST-GCaMP6f mouse. (**f** to **h**) Same as in (b) to (d), but for ChR2-SST-mCherry interneurons (n = 7 cells, 2 mice). The mean frequency rate was 1.74 ± 1.01 Hz at rest (spontaneous activity), 73.1 ± 13.1 Hz during the pulse and 13.4 ± 2.41 Hz during the overall stimulation period.

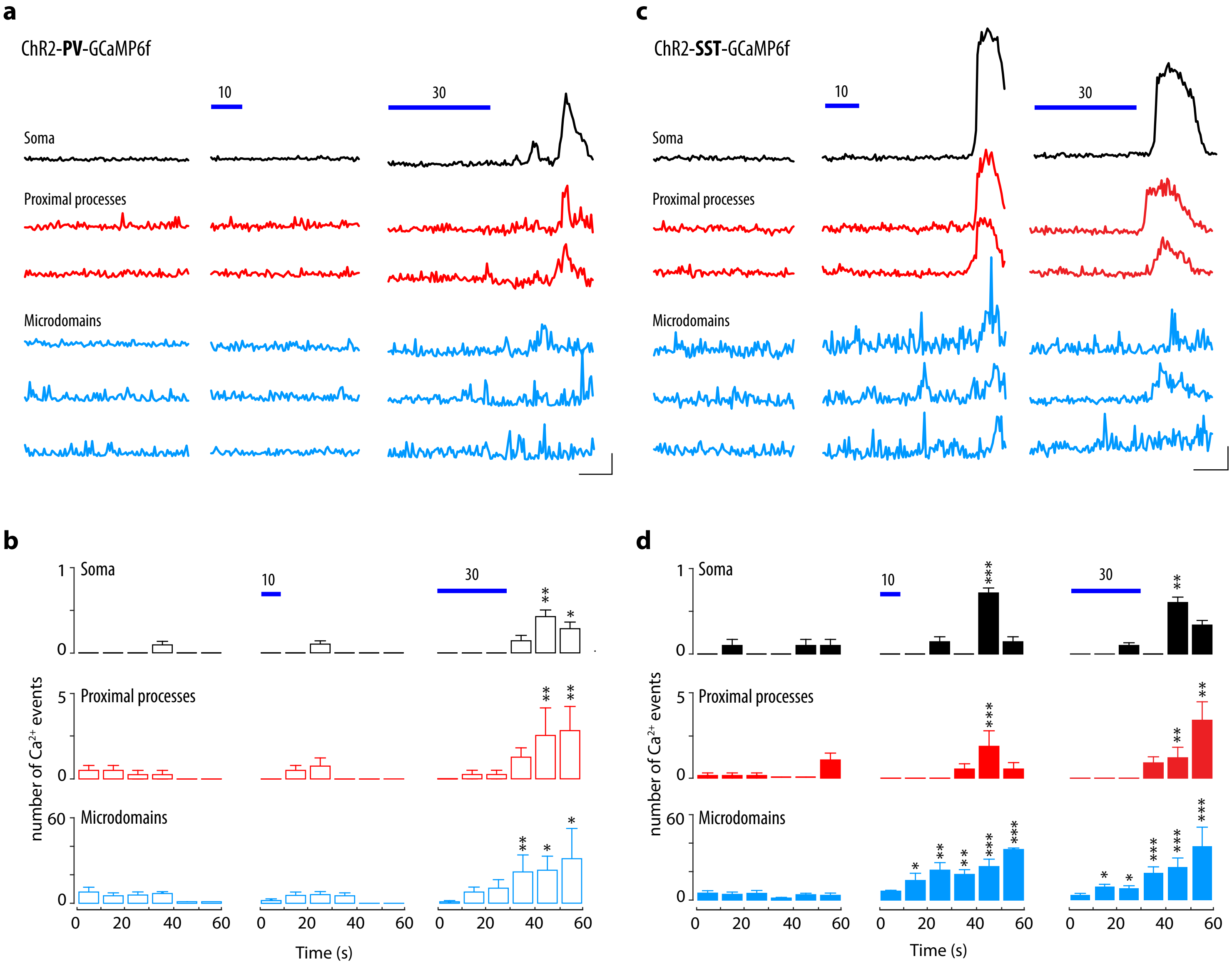

Supplementary Figure 4. PV and SST interneuron signaling fails to rapidly induce astrocytic Ca^{2+} elevations in SSCx slices. (**a**) Representative traces of Ca^{2+} signal dynamics at soma, proximal processes and microdomains of a representative GCaMP6f astrocyte in brain slice from a ChR2-PV-GCaMP6f mouse, before and during light pulse stimulation of ChR2-PV interneurons. Imaging was performed with confocal laser microscopy. Scale bars, 10 s and 50% dF/F0. (**b**) Mean number of Ca^{2+} events plotted in 10 s bins before, during and after 10 and 30 light pulse activation of ChR2-PV-GCaMP6f mice (11 astrocytes, 4 slices, 2 mice). Data are represented as mean ± SEM. (**c** and **d**) Same as in (a and b) but in slices from ChR2-SST-GCaMP6f mice (7 astrocytes, 7 slices, 3 mice).

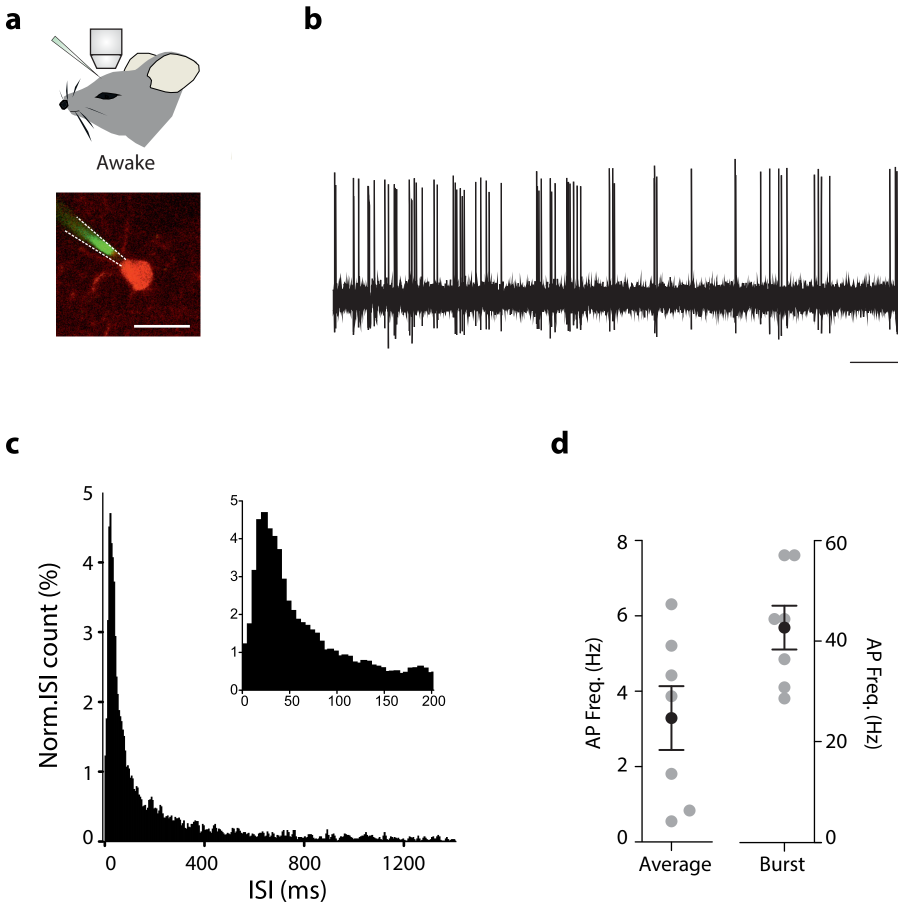

Supplementary figure 5. Two-photon-guided juxtasomal recordings from SST interneurons in the SSCx of awake mice. (**a**) Top, schematic drawing of the experimental setup for two-photon guided juxtasomal recordings in awake head-fixed mice. Bottom, two-photon image of a tdTomato (red) expressing layer 2/3 SST interneuron in SSCx. The neuron was targeted for juxtasomal recordings with a patch-pipette (dotted lines) filled with Alexa 488 (green). Scale bar, 20 μm. (**b**) Representative electrophysiological recording from the neuron represented in (a) showing the spontaneous action potential firing in a 10 s time window. Scale bars, 1 s, 0.2 mV. (**c**) Average (over cells) distribution of the interspike-intervals (ISI) in SST interneurons. Bin, 5 ms. The distribution is shown at an expanded time scale in the inset. (**d**) Left, mean firing frequency of layer 2/3 SST interneurons averaged over long time periods (range, 96 -540 s). Right, mean firing frequency of layer 2/3 SST interneurons during bursts, calculated as the reciprocal of the peak of the ISI distribution. The black dots represent the average of all experiments (gray dots; 7 cells, 3 mice). Data are represented as mean ± SEM.

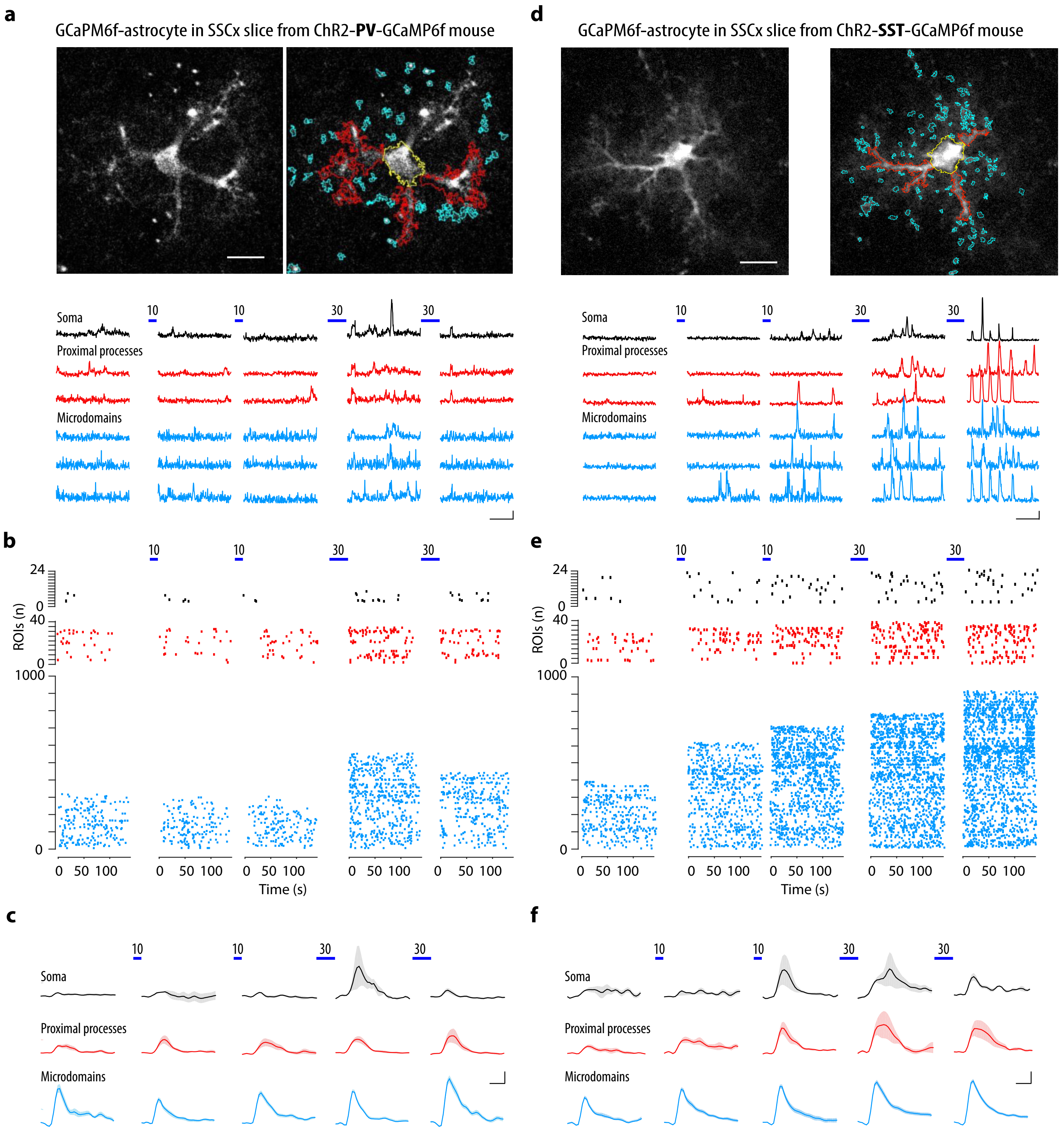

Supplementary Figure 6. Ca^{2+} signal imaging from SSCx slices confirms differential responses of astrocytes to PV and SST interneuron activation. (**a**) two-photon images (top) and representative Ca^{2+} signal dynamics (bottom) at soma, proximal processes and microdomains of a GCaMP6f-astrocyte in layer 2/3 SSCx slice from a ChR2-PV-GCaMP6f mouse, before and after successive light pulse PV interneuron activations. Scale bars, 20 µm, 50 s and 20% dF/F_0 . (**b**, **c**) Raster plots of Ca^{2+} peaks (b) and mean time course of Ca^{2+} transients (c) from GCaMP6f-astrocytes in SSCx slices, at rest and following PV interneuron stimulations for soma, proximal processes and microdomains. Scale bars, 5 s, 20% dF/F_0. (**d** to **f**) As in (a) to (c) but in slices from ChR2-SST-GCaMP6f mice.

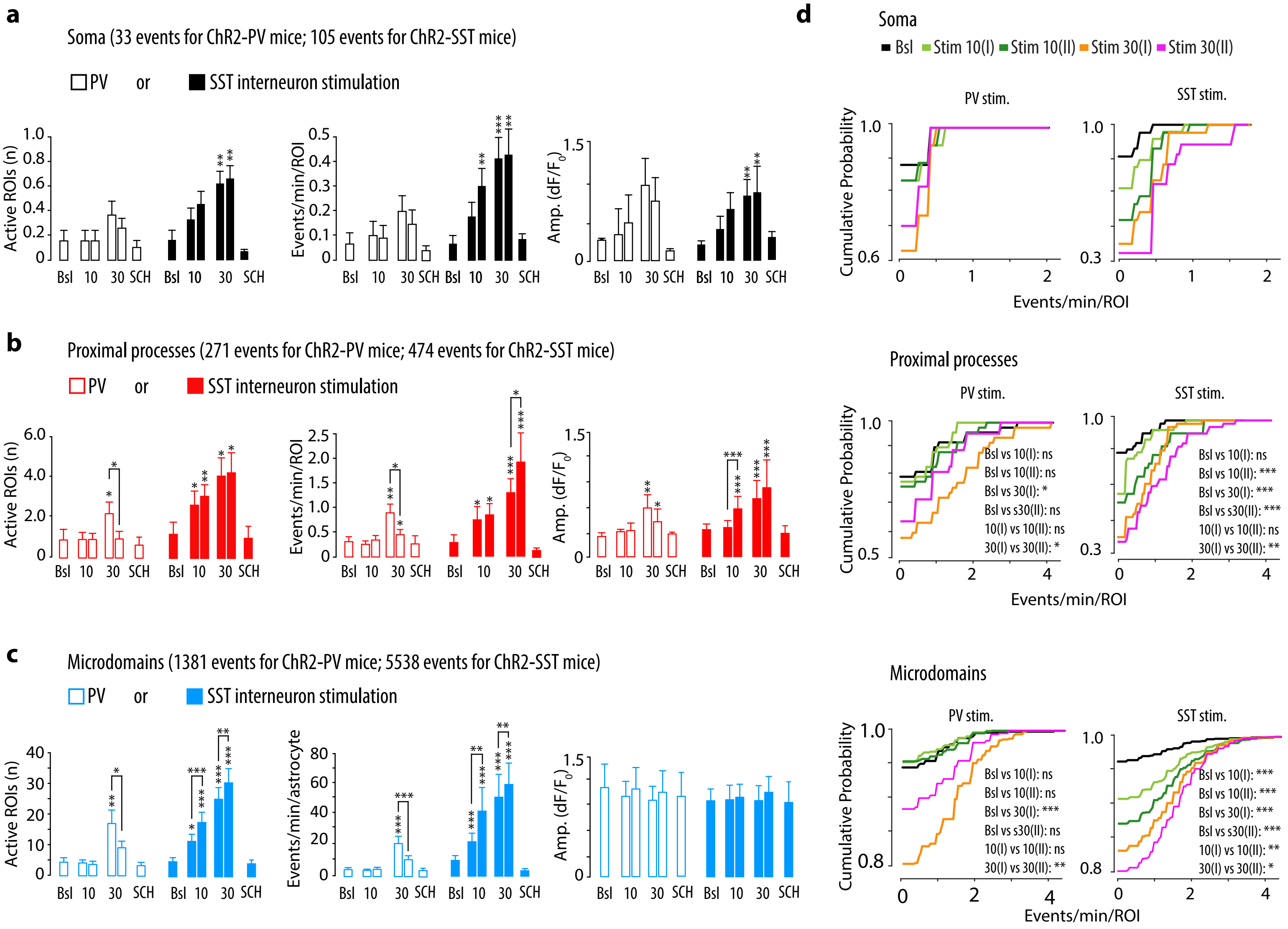

Supplementary Figure 7. Properties of astrocyte responses to PV and SST interneurons in brain slices. (**a** to **c**) Average data for the properties of the astrocyte Ca^{2+} response to PV interneuron (16 GCaMP6f-astrocytes, 12 slices, 5 mice) and SST interneuron signaling (19 GCaMP6f-astrocytes, 15 slices, 8 mice). SCH50911 (SCH, 50 μM) effect was evaluated on the first 30 light pulse stimulation of PV or SST interneurons (7 astrocytes, 7 slices from 3 mice for ChR2-PV mice; 11 astrocytes, 5 slices from 3 mice for ChR2-SST mice) . Data are represented as mean ± SEM. (**d**) Cumulative distributions of astrocytic Ca^{2+} events confirm significant response depression to successive PV interneuron stimulations and potentiation to successive SST interneuron stimulations for both proximal processes and micro-domains. * $p \leq 0.05$, ** $p \leq 0.01$; * $p \leq 0.001$; ns, not statistically different, Kolmogorov-Smirnov test.

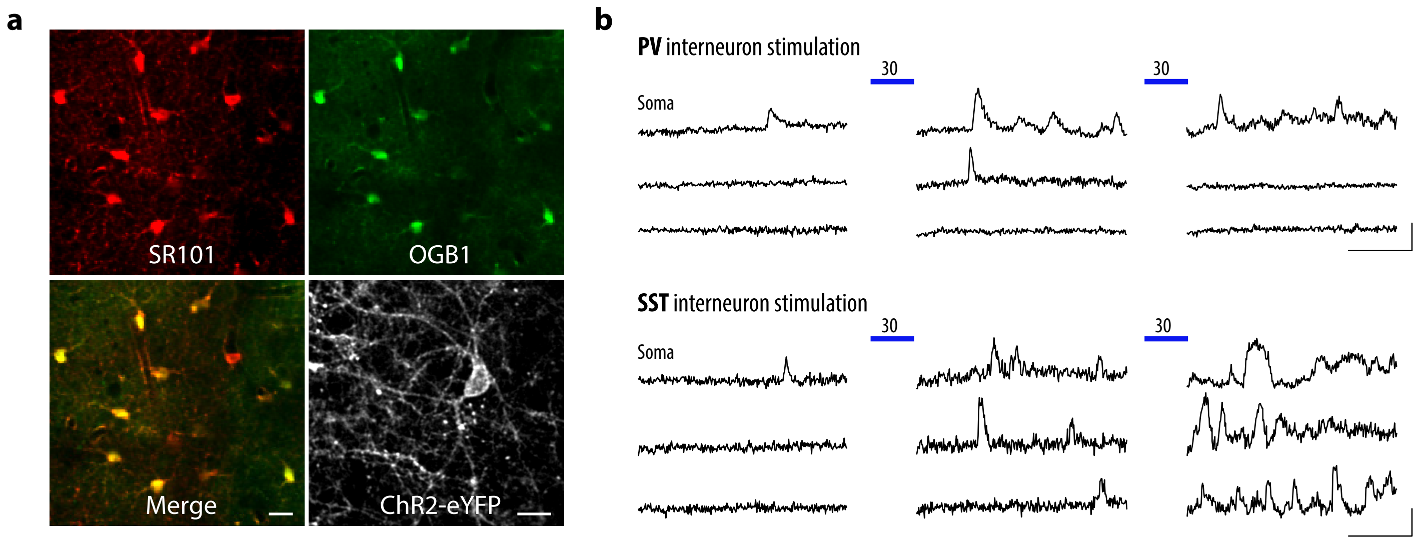

Supplementary Figure 8. PV and SST interneurons differentially recruit the astrocytic network in *in vivo* SSCx. (a) 2-photon images of layer 2/3 SSCx slices from a ChR2-PV mouse illustrating SR101-loaded astrocytes, OGB1-loaded astrocytes and the merged image. Also reported is an image of a ChR2-expressing EYFP-labeled PV interneuron. Scale bar, 25 µm. **(b)** Somatic Ca^{2+} signal dynamics from representative in vivo astrocytes before and after two sequences of 30 light pulse activation of PV or SST interneurons. Scale bar, 50 s, 20% dF/F0.

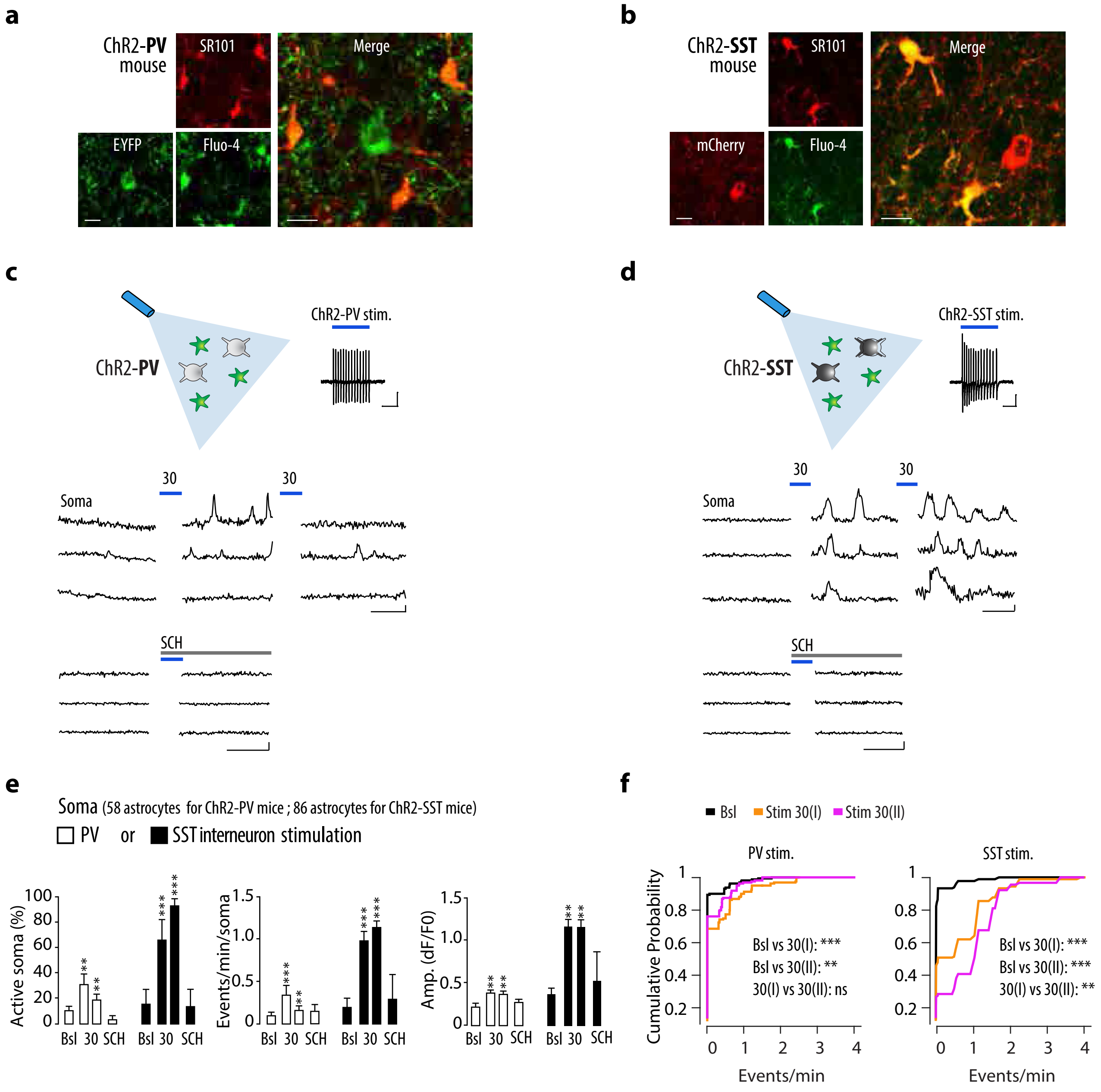

Supplementary Figure 9. PV and SST interneurons differentially recruit the astrocytic network in SSCx slices. (**a** and **b**) Two-photon images of layer 2/3 SSCx slices from a ChR2-PV (**a**) or a ChR2-SST (**b**) mouse illustrating SR101-loaded astrocytes, Fluo-4-loaded astrocytes, ChR2-expressing EYFP-labeled PV interneurons (a) or mCherry-labeled SST interneurons (b), and merged signals. Scale bars, 10 µm. (**c** and **d**) Top, schematics of optogenetic experiments and AP firing in juxtasomal recordings from a ChR2-PV (c) or ChR2-SST interneuron (d), evoked by a single light pulse. Scale bars: 0,5 mV, 50 ms. Bottom, somatic Ca^{2+} signal dynamics from representative astrocytes, before and after two sequences of 30 light pulse activation of PV (c) or SST interneurons (d). The effect of SCH50911 (SCH, 50 µM) was evaluated on the first 30 light pulse stimulation of PV or SST interneurons. Scale bars, 50 s, 20% dF/F_0. (**e**) Mean percentage of responsive astrocytes and mean Ca^{2+} event frequency in response to PV interneuron (white bars, 58 astrocytes, 4 slices, 2 mice) or SST interneuron (black bars, 86 astrocytes, 5 slices, 3 mice) signaling. Data are represented as mean ± SEM. (**f**) Cumulative distributions of Ca^{2+} event frequency after two subsequent 30 light pulse activations. Notably, with respect to the first stimulation, the astrocyte response to the second PV interneuron stimulation decreased, although not significantly, whereas that to the second SST interneuron stimulation significantly increased (Kolmogorov-Smirnov test, $**p \leq 0.01$, $*** p \leq 0.001$).

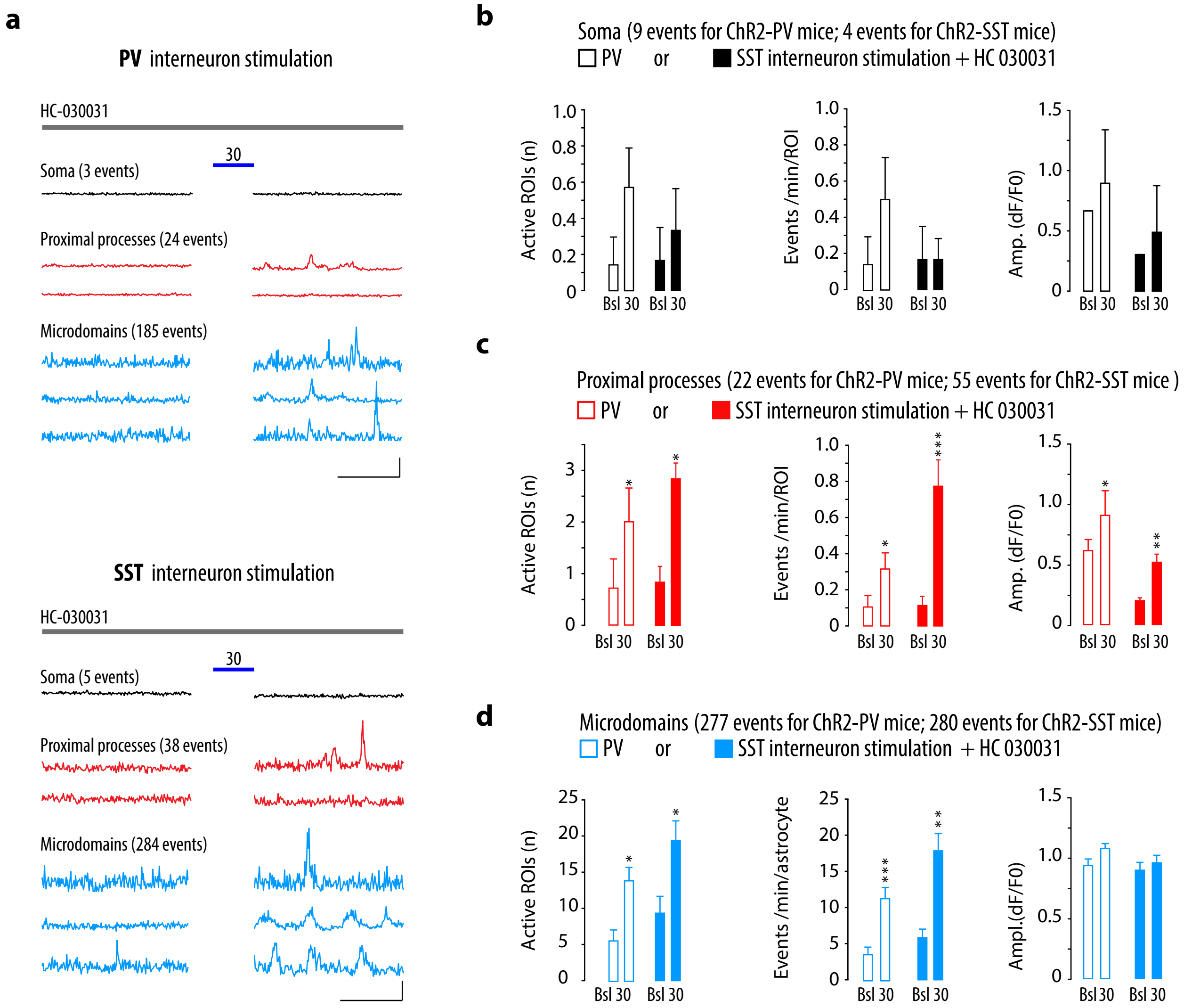

Supplementary Figure 10. Astrocyte responses to PV and SST interneurons do not depend on TRPA1 channel activation. (**a**) Representative Ca^{2+} signals in soma, proximal processes and microdomains of GCaMP6f-astrocytes from slice preparations in response to PV or SST 30 pulse stimulation in the presence of the TRPA1 antagonist HC 030031 (80 µM). Scale bars, 50 s, 50% dF/F_0 (**b** to **d**) Average data for the properties of astrocyte Ca^{2+} response to PV or SST interneuron activation (for PV interneurons, 6 astrocytes, 6 slices; 2 mice; for SST interneurons, 6 astrocytes, 5 slices, 2 mice). Data are represented as means ± SEM.

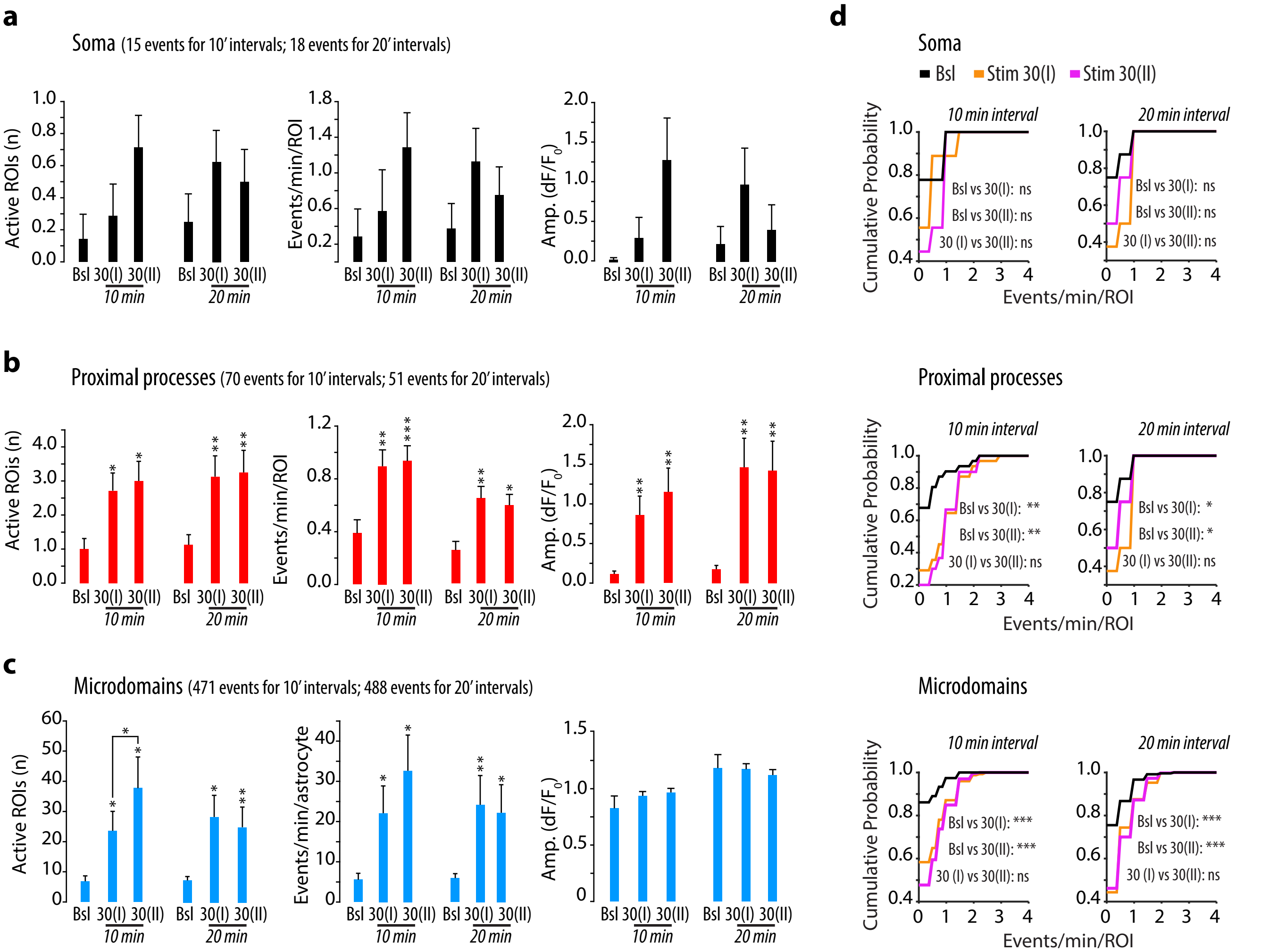

Supplementary Figure 11. Time window of astrocyte response potentiation to SST interneurons. Average data for the properties of the astrocyte Ca^{2+} response to SST interneurons comparing two successive stimuli applied with 10 or 20 min intervals (7 GCaMP6f-astrocytes, 7 slices , 4 mice, for 10 min interval and 8 GCaMP6f-astrocytes, 8 slices, 5 mice for 20 min interval experiments). Data are represented as mean ± SEM. (**d**) Cumulative distributions of astrocytic Ca^{2+} events reveal significant response potentiation with 10 min interval in microdomains but not in other compartments, while no potentiation was observed with 20 min interval. * $p \leq 0.05$, ** $p \leq 0.01$; * $p \leq 0.001$; ns, not statistically different, Kolmogorov-Smirnov test.

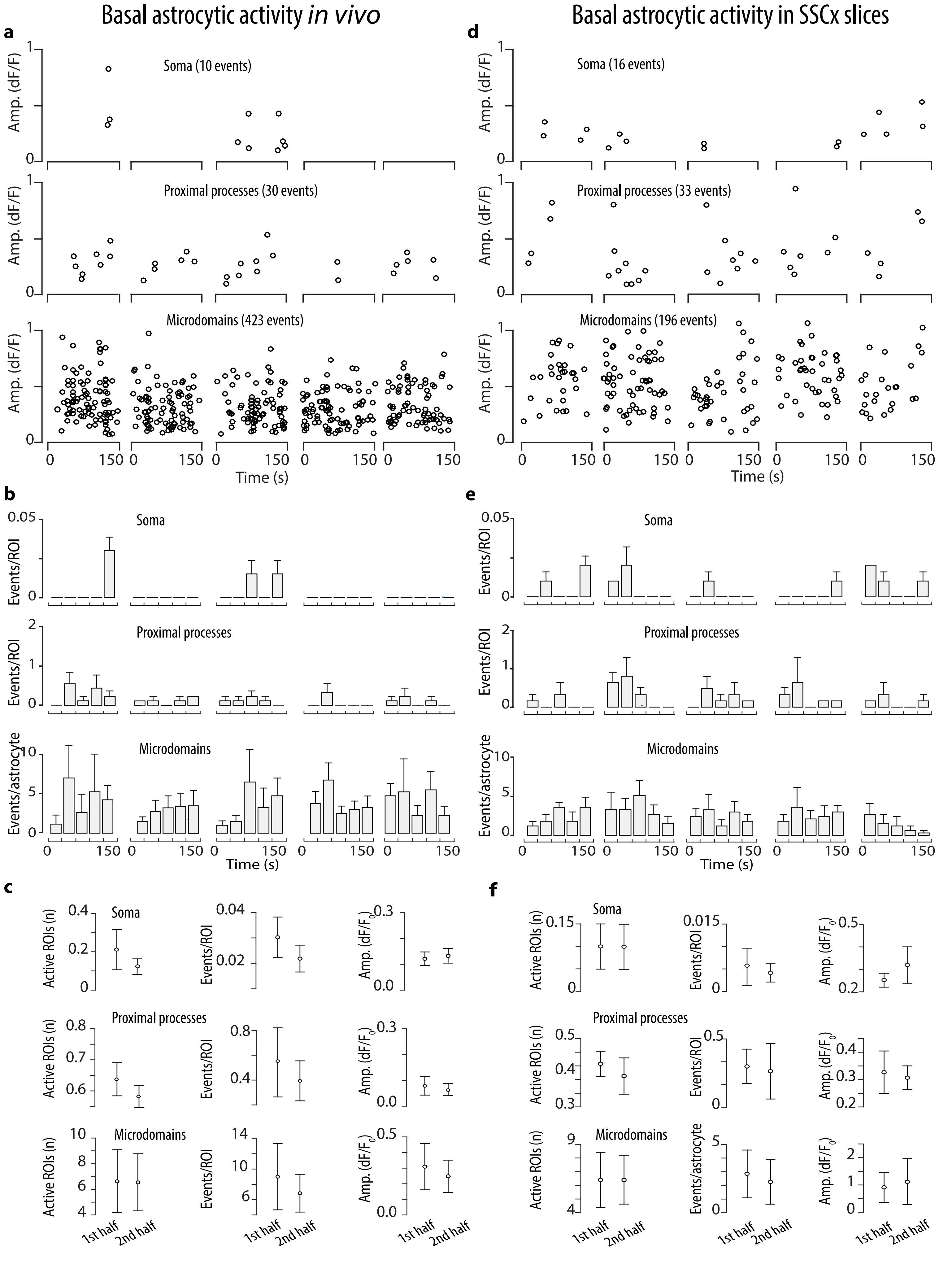

Basal astrocytic activity in vivo
Basal astrocytic activity in SSCx slices
a
d
Soma (10 events)
Proximal processes (30 events)
Microdomains (423 events)
Soma (16 events)
Proximal processes (33 events)
Microdomains (196 events)
Amp. (dF/F)
Time (s)
b
e
Soma
Proximal processes
Microdomains
Events/ROI
Events/astrocyte
c
f
Active ROIs (n)
Events/ROI
Events/astrocyte
Amp. (dF/F0)
1st half
2nd half

Supplementary Figure 12. Successive 2P laser acquisitions do not evoke astrocyte Ca^{2+} responses.

(**a**) Amplitude of Ca^{2+} events measured during in vivo experiments, plotted as a function of time and pooled from all astrocytes (11 astrocytes, 2 mice) recorded in the soma, proximal processes and microdomains during 5 successive 2P laser scanning acquisitions with same duration (150 s), same inter-acquisition interval and laser parameters used in this study. (**b**) Histograms showing the average number of spontaneous Ca^{2+} events for each imaging session, analyzed in 30 s time bins. Data are represented as mean ± SEM. (**c**) Average number of active ROIs (left), frequency (middle) and event amplitudes (right) during the first and second half of the recording. Data were analyzed for astrocyte somas, proximal processes and microdomains. No significant variation was observed between the two halves of the recordings. (**d-f**) Same as in a-c for experiments in slice preparations (18 astrocytes, 7 slices, 3 mice).

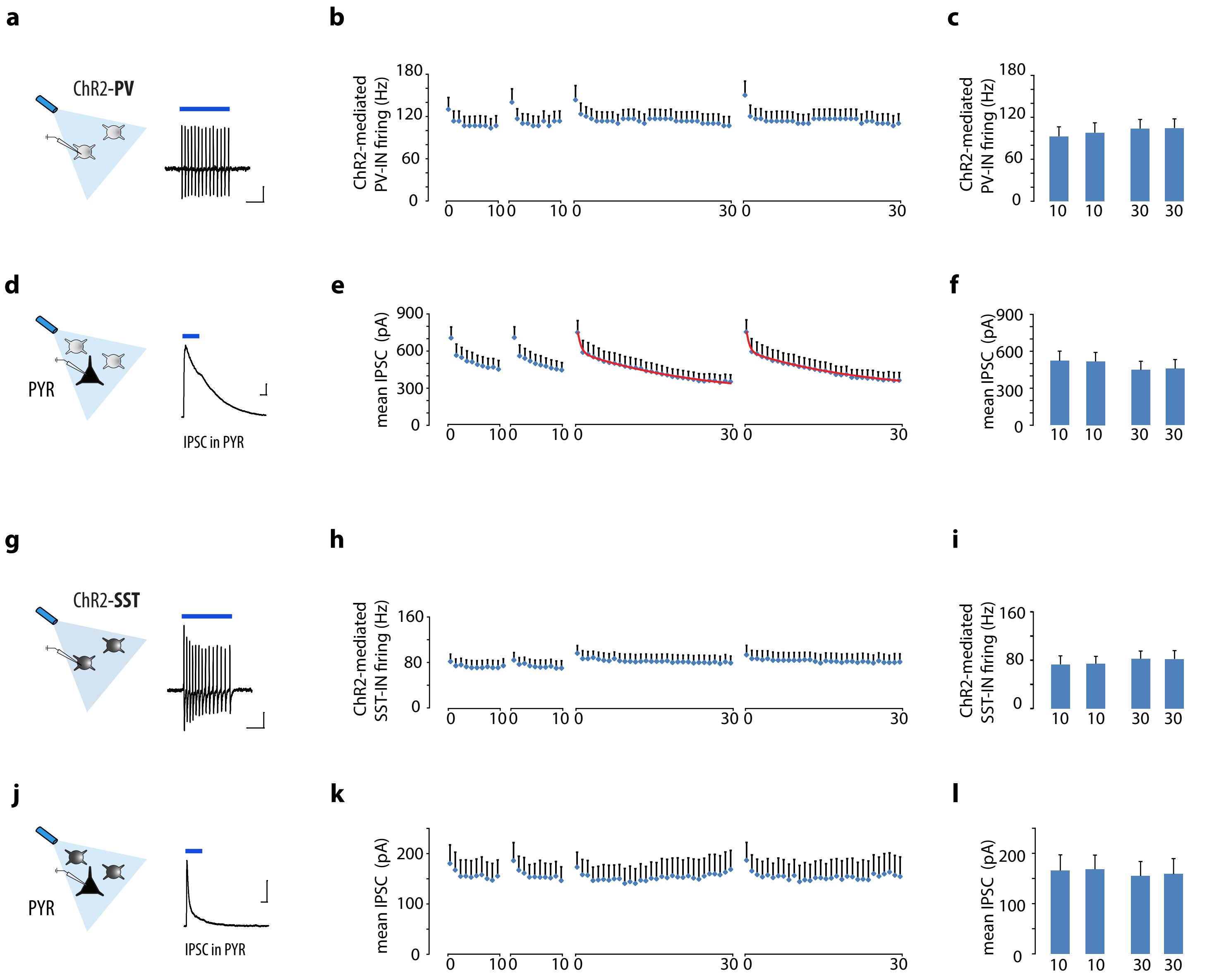

Supplementary Figure 13. Unchanged firing rates in ChR2-PV or ChR2-SST interneurons and unchanged IPSCs in pyramidal neurons during successive optogenetic stimulations. (**a**) Schematic of the experiment and representative juxtaposed recordings of AP firing discharge from a ChR2-PV interneuron upon light activation in SSCx slices from a ChR2-PV mouse. Scale bars, 50 ms, 0.5 mV. (**b-c**) Average firing rate from ChR2-PV interneurons (n = 8, 8 slices, 3 mice) reported for each light pulse (b) or as the mean of 10 or 30 pulses (c). (**d**) Schematic of the experiment and representative evoked IPSCs recorded from a pyramidal neuron upon light activation in SSCx slices from a ChR2-PV mouse. Scale bars, 50 ms, 100 pA. (**e-f**) Average IPSC peak amplitude recorded from pyramidal neurons (n = 7, 4 slices, 2 mice) for each light pulse (e) or as the mean of 10 or 30 pulses (f). Mean IPSC peak amplitudes were fitted (red lines in e) to double exponential equation (see Methods). For the first 30 pulse stimulus decay time constants of the fast and slow components were, respectively, 0.23 s and 18.26 s (R^2, 0.994); for the second 30 pulse stimulus decay time constants were 0.34 s and 19.06 s (R^2, 0.994). The 30th IPSC mean peak amplitude normalized to the first was 0.46 ± 0.03 for the first 30 pulse stimulus and 0.47 ± 0.04 for second. (**g-i**) Same as in (a-f), but in slices from ChR2-SST mice (n = 8, 6 slices, 3 mice for SST interneuron firing and n = 8, 6 slices, 4 mice for IPSCs in pyramidal neurons). Data are represented as mean ± SEM.

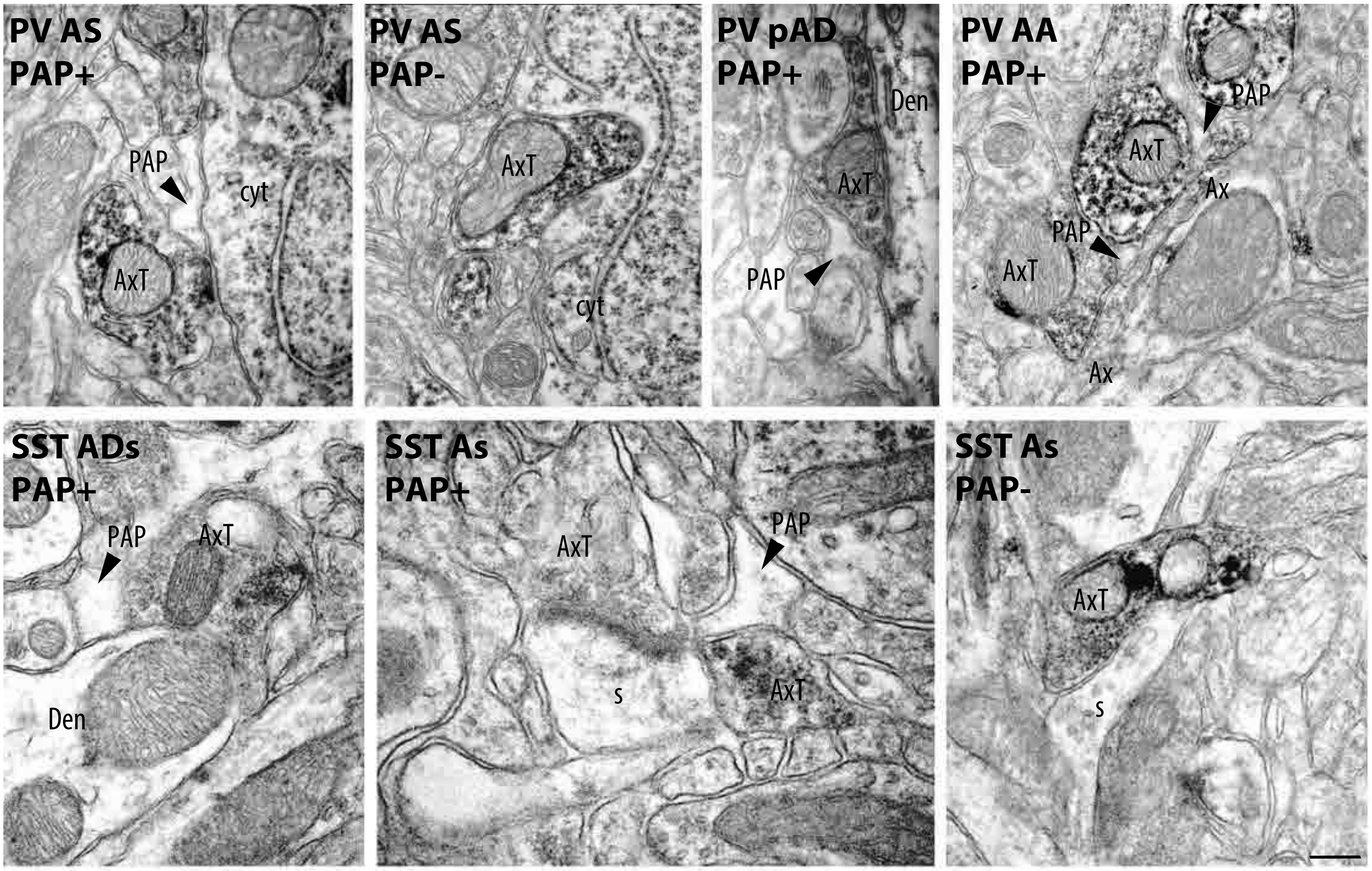

Supplementary Figure 14. SST and PV synapses related to perisynaptic astrocytic processes (PAPs) are similarly distributed. Pre-embedding electron microscopy of PV and SST interneuron positive synapses from immunoperoxidase material. **Upper row**, examples of PV positive terminals forming synapses on neuron somata (PV axo-somatic (AS) synapses). In one case, such terminal is associated with a PAP (PAP+), whereas in the other case it was not (PAP-). PV positive axon terminals to a proximal axo-dendritic (pAD) and axo-axonic (AA) synapses associated with PAP are also shown. **Lower row**, examples of a SST positive terminal on an axo-dendritic shaft (ADs) associated with a perisynaptic astrocytic process (PAP+), of a SST positive terminal of an axo-spinous (As) synapse (where spine also receives an asymmetric synaptic contact on the top) associated with PAP and of a SST positive terminal of an As not associated with PAP. AxT, axon terminal; cyt, cytoplasm; Den, dendrite; Ax, axon; s, spine. Scale bar: 200 nm.

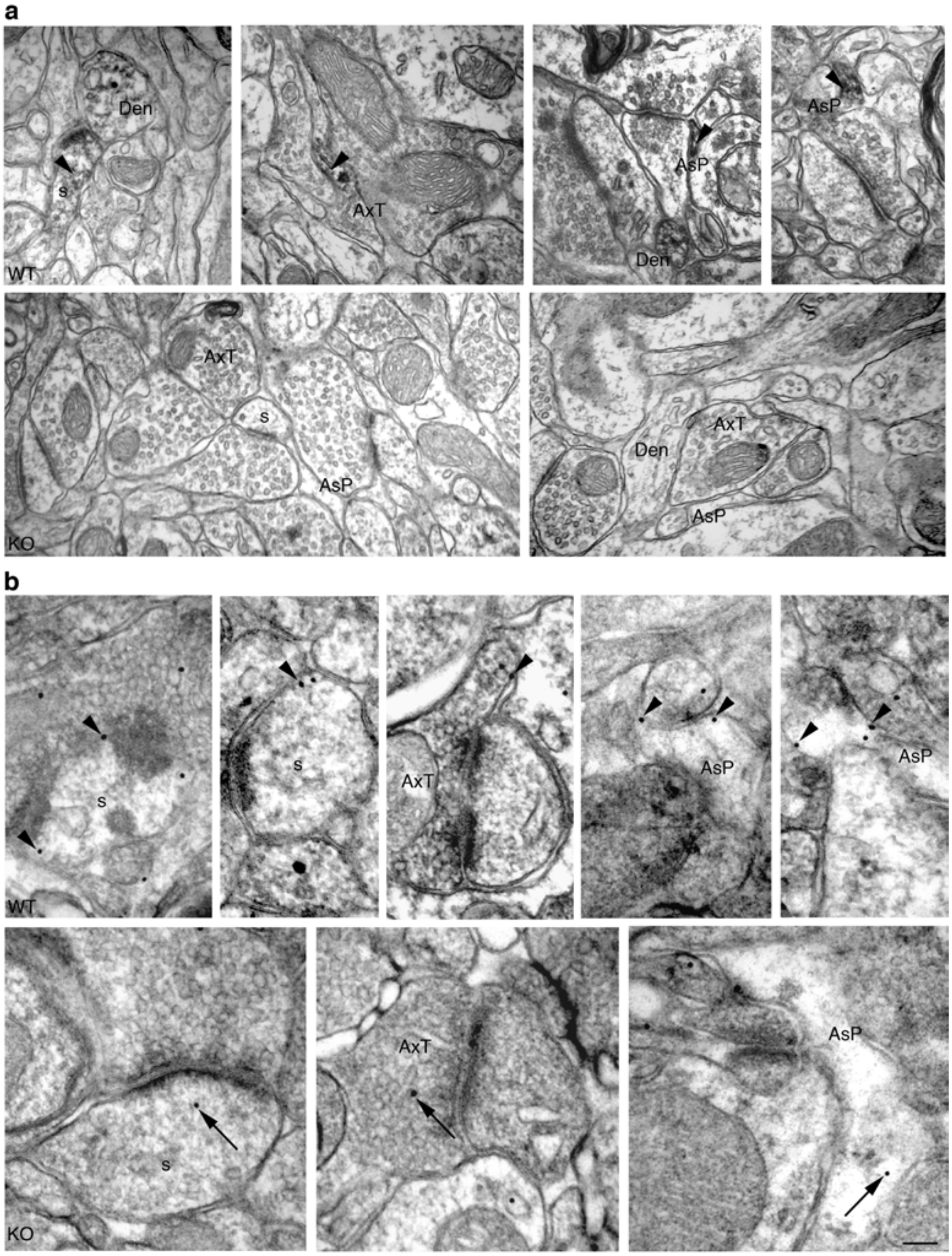

Supplementary Figure 15. Specificity of anti-GABA$_{B2}$ mouse monoclonal antibody: pre- and post-embedding GABA$_{B2}$ KO studies. Pre- and post-embedding electron microscopy from the neocortex of WT and GABA$_{B2}$ KO mice revealed the specificity of GABA$_{B2}$ detection by the primary anti-GABA$_{B2}$ mouse monoclonal antibody (sc-393270, Santa Cruz Biotechnology Inc., USA). (**a**) pre-embedding electron microscopy of immunoperoxidase material from WT and GABA$_{B2}$ KO mice. Upper row, in WT dense immunoreactive product (*arrowheads*) was detectable in spines (s), dendrites (den), axon terminals (AxT), and astrocytic processes (AsP). Lower row, in all compartments of GABA$_{B2}$ KO, GABA$_{B2}$ immunoreactivity was not observed. Tissue preparation, immunoperoxidase, and pre-embedding method were described in Methods. GABA$_{B2}$ primary antibody dilution was 1:50. Data are from the most superficial ultrathin sections (15 sections/animal) from layers 2/3 of SSCx. (**b**) Post-embedding electron microscopy of immunogold reacted material from WT and GABA$_{B2}$ KO mice. Upper row, in WT mice 18 nm GABA$_{B2}$-coding particles were preferentially detectable at the membranes of spines (s), AxT and AsP (*arrowheads*). In KO mice (lower row), sparse colloidal gold particles (*arrows*) were detectable in the cytoplasm and almost absent at membranes. For quantification and statistical analysis of immunogold staining, see supplementary Table 2. See Methods for tissue preparation, post-embedding method (including primary antibodies dilution), and density analysis. Data are from layers 2/3 of SSCx. Scale bar: 150 nm for panel a, and 100 nm for panel b.

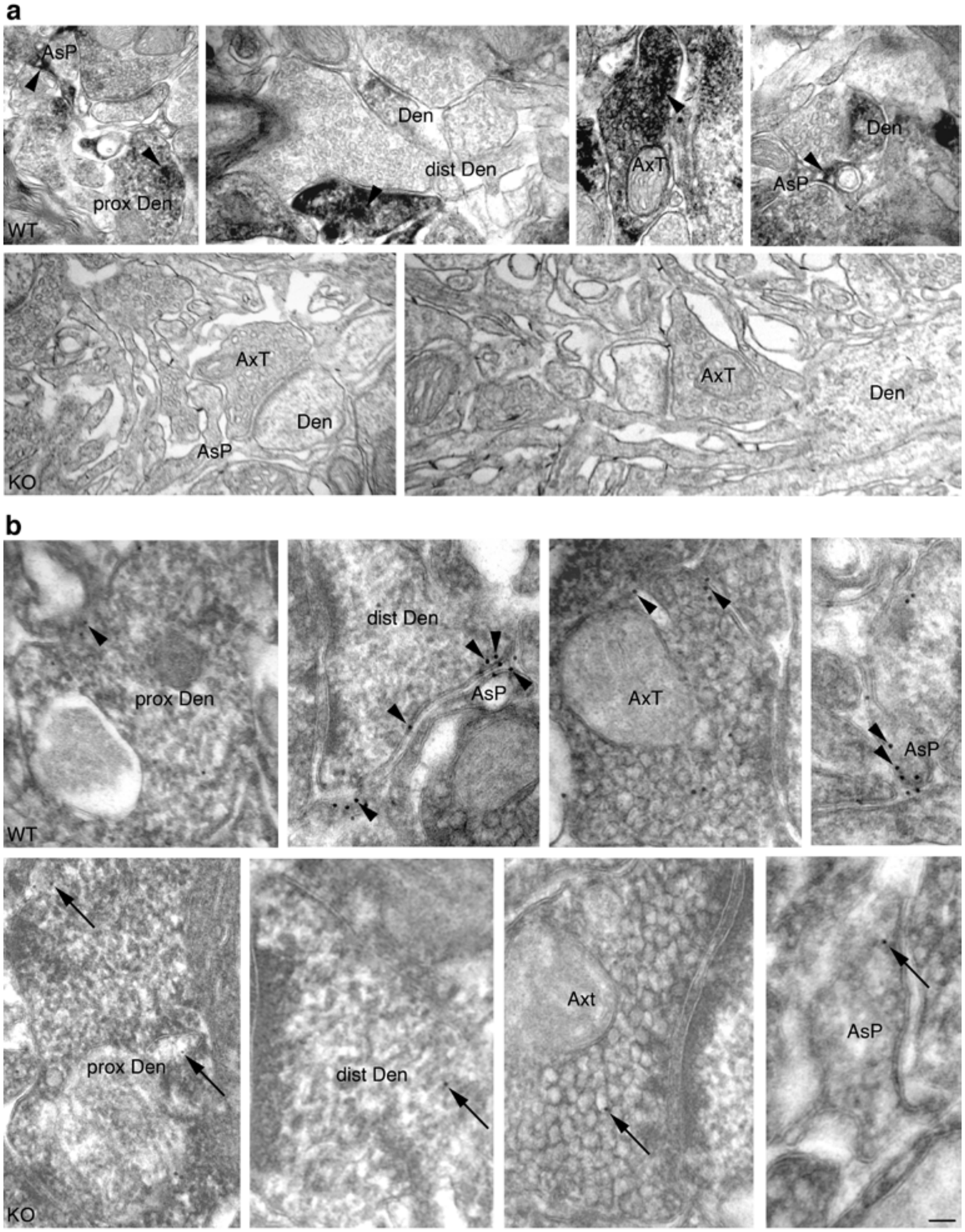

Supplementary Figure 16. Specificity of anti-SSTR4 rabbit polyclonal antibody: pre- and post-embedding SSTR4 KO studies. Pre- and post-embedding electron microscopy from the neocortex of WT and SSTR4 KO mice revealed the specificity of SSTR4 detection by the primary anti-SSTR4 rabbit polyclonal antibody (sc-25678, Santa Cruz Biotechnology Inc., USA). (**a**) pre-embedding electron microscopy of immunoperoxidase material from WT and SSTR4 KO mice. Upper row, in WT dense immunoreactive product (*arrowheads*) was detectable in proximal dendrites (prox Den), astrocytic processes (AsP), distal dendrites (dist Den), and axon terminals (AxT). Lower row, in all compartments of SSTR4 KO SSTR4 immunoreactivity was not observed. Tissue preparation, immunoperoxidase, and pre-embedding method were described in Material and Methods. SSTR4 primary antibody dilution was 1:50. Data are from the most superficial ultrathin sections (15 sections/animal) of layers 2/3 of SSCx. (**b**) Post-embedding electron microscopy of immunogold reacted material from WT and SSTR4 KO mice. Upper row, in WT mice 12 nm SSTR4-coding particles were preferentially detectable at the membranes of prox Den, dist Den, AxT and AsP (*arrowheads*). In KO mice (lower row), sparse colloidal gold particles (*arrows*) were detectable in the cytoplasm and almost absent at membranes. For quantification and statistics of immunogold staining, see supplementary Table 3. Post-embedding method (including primary antibodies dilution), and density analysis are described in Methods. Data are from ultrathin sections (12 sections/animal) of layers 2/3 of SSCx. Scale bar: 300 nm for the proximal dendrite of upper row, 150 nm for all the other fields of panel a, 130 nm for proximal dendrites of panel b, and 100 nm for all the other fields of panel b.

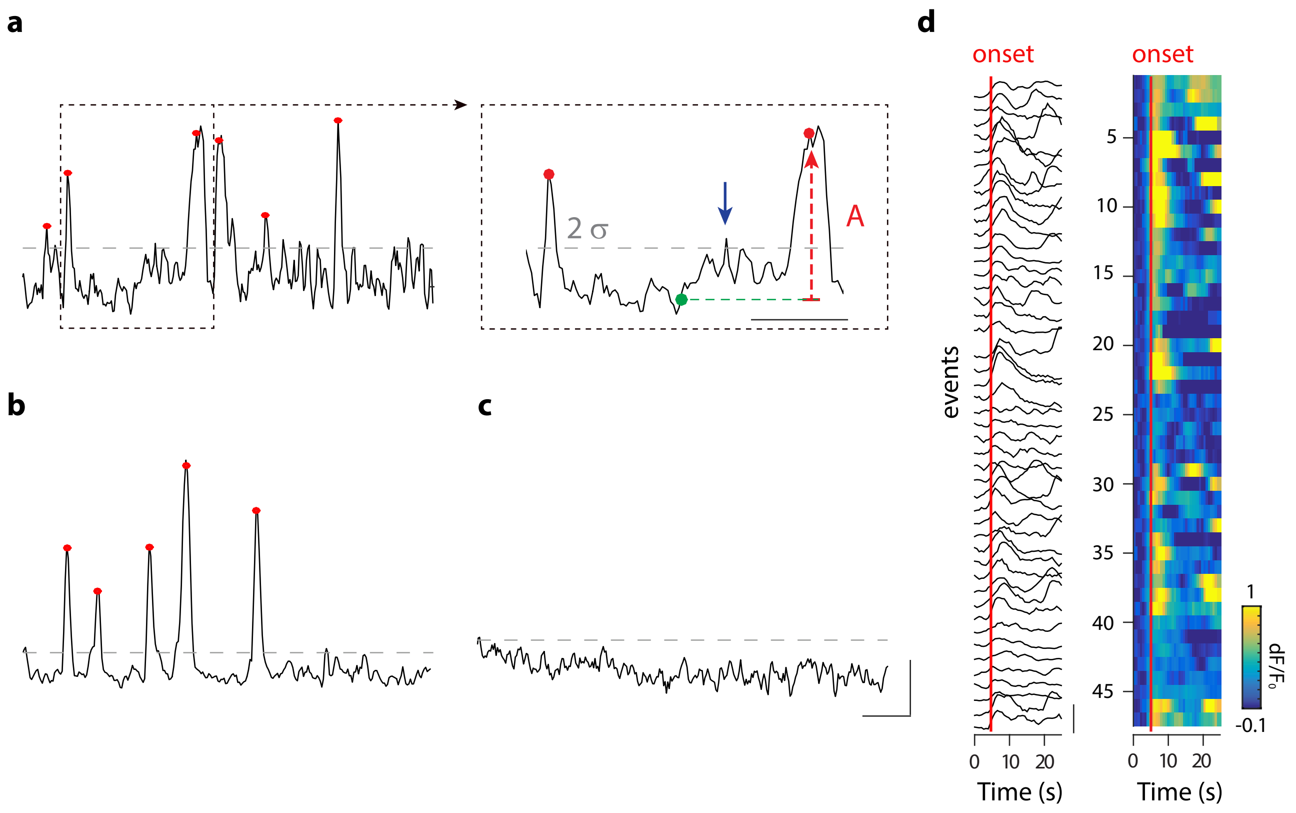

Supplementary Figure 17. Detection and analysis of Ca^{2+} events from GCaMP6f astrocytic processes (**a**) Examples of a dF/F_0 trace over time from a GCaMP6f astrocytic process. Red dots indicate significant Ca^{2+} events that were detected through a procedure which is illustrated synthetically in the dashed line panel (right) and described in detail in the Methods. The grey dashed line represents the threshold of 2 SDs (σ) of the *baseline trace*. We considered significant only the local maxima that crossed this fixed threshold (red dots) and have a total amplitude (a) greater than 3 SDs of the *baseline trace*. Amplitude of the peak was computed from the 20th percentile of the trace interposed between the current and the previous peak (green dots). Essentially, this procedure combines a threshold measured from the global baseline with a stricter threshold computed from a local baseline. Blue arrow indicates a Ca^{2+} peak that was rejected because it crossed the 2 SDs threshold only. (**b**, **c**) Other representative examples of a trace with multiple peaks that were all accepted (b) and a trace where no Ca^{2+} peak was detected (c). Scale bars, 20 s and 1dF/F_0. (**d**) Left panel, Ca^{2+} events detected in all ROIs of the proximal processes from an individual astrocyte, aligned to onset (red line). Onset was defined as the last point in the dF/F_0 timecourse below 1 SD before a significant peak occurred. Right panel, dF/F_0 calculated for each Ca^{2+} peak detected.

number (n) of synapses	*% of synapses associated with PAP*	*% of synapses with monolateral PAP*	*% of synapses with bilateral PAP*
**PV** (n=380)	62.11 ± 2.49§	88.51 ± 2.08	11.49 ± 2.08
AS (n=155)	61.94 ± 3.91°	86.32 ± 3.54	13.18 ± 3.54
pAD (n=120)	66.67 ± 4.32°	91.25 ± 3.17	8.75 ± 3.17
AA (n= 105)	57.14 ± 4.85°	88.33 ± 4.17	11.67 ± 4.17
**SST** (n=296)	68.58 ± 2.70§	89.66 ± 2.14	10.34 ± 2.14
ADs (n=145)	64.83 ± 3.97°	95.74 ± 2.09	4.26 ± 2.09
As (n=151)	72.19 ± 3.65°	84.40 ± 3.49	15.60 ± 3.49

Supplementary Table 1. Quantification of PAPs at symmetric synapses formed by axon terminals from PV or SST interneurons.

In line with the notion that PV and SST interneuron positive (PV+ and SST+) terminals form symmetric synapses with different postsynaptic targets, the identification of perisynaptic astrocytic processes was performed at axo-somatic, proximal axo-dendritic and axo-axonic synapses for PV+ interneuron terminals, and at axo-dendritic shaft and axo-spinous synapses for SST+ interneuron terminals. Quantitative analysis of the frequency of perisynaptic astrocytic processes did not reveal differences between PV and SST interneuron synapses. § Mann-Whitney test did not reveal differences between the percentages of PV+ and SST+ interneuron synapses related to PAPs ($P = 0.08$). Data from layers 2/3 of SSCx of pre-embedded immunoperoxidase materials from three mice (15 ultrathin sections/animal/antigen). Distribution of PAPs at axo-somatic (AS), proximal axo-dendritic (pAD) and axo-axonic (AA) PV+ synapses and at axo-dendritic shaft (ADs) and axo-spinous (As) SST+ synapses is also reported. °Kruskall Wallis with Dunn's multiple comparison test reveal no differences between subgroups of PV+ and SST+ synapses (AS vs pAD, $P > 0.99$; AS vs AA, $P > 0.99$; AS vs ADs, $P > 0.99$; AS vs As, $P = 0.60$; pAD vs AA, $P > 0.99$; pAD vs ADs, $P > 0.99$; pAD vs As, $P > 0.99$; AA vs ADs, $P > 0.99$; AA vs As, $P = 0.13$; ADs vs As; $P > 0.99$).

Localization	*WT*	*KO*	*WT vs KO*
**Pyramidal nuclei**			
area (μm^2)			
mean ± SEM	48.59 ± 4.46 (n=20)	48.59 ± 4.95 (n=20)	P=0.989
median	44.03	43.77	
background density (particles/μm^2)			
mean	0.73 ± 0.12°°	0.66 ± 0.05§	P=0.289
median	0.49	0.71	
**Spines**			
area			
mean ± SEM	0.35 ± 0.03 (n=58)	0.38 ± 0.03 (n=62)	P=0.657
median	0.30	0.32	
t density			
mean	6.22 ± 0.63	4.91 ± 0.49	P=0.021 (*)
median	5.23	3.76	
mem density			
mean	20.15 ± 2.75°°	9.16 ± 1.69§	P=0.001 (**)
median	17.70	0	
**Axon terminals**			
area			
mean ± SEM	0.82 ± 0.09 (n=62)	0.74 ± 0.05 (n=60)	P=0.688
median	0.61	0.57	
t density			
mean	3.28 ± 0.34	2.73 ± 0.22	P=0.249
median	2.66	2.24	
mem density			
mean	13.17 ± 1.52°°	4.90 ± 1.16§	P<0.0001 (****)
median	14.19	0	
**Astrocytic processes**			
area			
mean ± SEM	0.33 ± 0.05 (n=54)	0.41 ± 0.04 (n=61)	P=0.091
median	0.18	0.29	
t density			
mean	11.23 ± 1.49	8.0 ± 1.40	P=0.015 (*)
median	7.76	3.94	
mem density			
mean	29.56 ± 3.51°°	10.09 ± 1.81§	P<0.0001 (****)
median	24.13	0	

Supplementary Table 2. Quantitative immunogold in $GABA_{B2}$ KO mice.

Quantitative analysis of $GABA_{B2}$ immunogold-labelling in spines, axon terminals (AxT) and astrocytic processes (AsP) in WT and KO mice validated the specificity of primary anti-$GABA_{B2}$ mouse monoclonal antibody (sc-393270, Santa Cruz Biotechnology Inc., USA). Data are from ultrathin sections (12 sections/animal) of SSCx layers 2/3. n, indicates number of profiles analyzed; t and mem density, indicate total and membrane-associated gold particles density, respectively. Column on the right (WT vs KO) reports comparisons between WT and KO (Mann-Whitney tests). Areas of all compartments sampled for quantitative analysis were comparable between WT and KO. With the exception of pyramidal nuclei (background) and t density of AxT, all t densities and mem densities of WT were significantly higher than those of KO (Mann-Whitney tests). °°, § comparison (Mann-Whitney tests) between densities of background and membrane-associated gold particles in WT (°°) and KO (§) mice. In spines, AxT and AsP of WT mice, densities of membrane-associated gold particles were significantly higher than in pyramidal nuclei (P=0.037, P=0.029, and P<0.0001, respectively) whereas in spines, AsP and AxT of KO mice were comparable to or relatively lower than background (P=0.068, P=0.739, and P=0.0005, respectively), showing that immunogold-labelling was virtually absent at the membranes of $GABA_{B2}$ KO mice.

Localization	*WT*	*KO*	*WT vs KO*
**Pyramidal nuclei**			
area (μm^2)			
mean ± SEM	33.24 ± 2.40 (n=10)	32.75 ± 2.01 (n=10)	P>0.999
median	31.76	30.01	
background density (particles/μm^2)			
mean	2.32 ± 0.11°°	1.98 ± 0.18§	P=0.075
median	2.24	1.94	
**Proximal dendrites**			
area			
mean ± SEM	1.96 ± 0.61 (n=13)	2.17 ± 0.38 (n=18)	P=0.275
median	1.23	1.90	
t density			
mean	5.00 ± 0.84	2.40 ± 0.37	P=0.007 (**)
median	4.55	1.72	
mem density			
mean	20.12 ± 4.25°°	3.68 ± 1.51§	P=0.0005 (***)
median	21.05	0	
**Distal dendrites**			
area			
mean ± SEM	0.45 ± 0.03 (n=75)	0.56 ± 0.05 (n=70)	P=0.316
median	0.43	0.44	
t density			
mean	8.81 ± 0.93	4.85 ± 0.38	P<0.0001 (****)
median	6.62	4.39	
mem density			
mean	20.31 ± 2.18°°	5.19 ± 1.20§	P<0.0001 (****)
median	16.39	0	
**Axon terminals**			
area			
mean ± SEM	0.58 ±0.05 (n=54)	0.73 ± 0.07 (n=50)	P=0.224
median	0.50	0.54	
t density			
mean	6.74 ± 0.75	3.79 ± 0.46	P=0.0004 (***)
median	5.39	2.67	
mem density			
mean	19.95 ± 2.39°°	4.53 ± 1.28§	P<0.0001 (****)
median	18.21	0	

**Astrocytic processes**<br>area<br>*mean ± SEM*<br>*median*	 <br>0.13 ± 0.01 (n=49)<br>0.10	 <br>0.14 ± 0.02 (n=34)<br>0.11	 <br>P=0.885
t density<br>*mean*<br>*median*	 22.76 ± 1.96<br>20	 17.28 ± 2.29<br>12.31	 P=0.019 (*)
mem density<br>*mean ± SEM*<br>*median*	 38.15 ± 3.36°°<br>31.25	 14.56 ± 4.33§<br>0	 P<0.0001 (****)

Supplementary Table 3. **Quantitative immunogold in SSTR4 KO mice.**
Quantitative analysis of SSTR4 immunogold-labelling in proximal (prox den) and distal (dist den) dendrites (dendrites were considered distal if their diameter was < or = 1μm, proximal if it was > 1μm), axon terminals (AxT) and astrocytic processes (AsP) in WT and KO mice validated the specificity of primary anti-SSTR4 rabbit polyclonal antibody (sc-25678, Santa Cruz Biotechnology Inc., USA). Data are from ultrathin sections (12 sections/animal) of SSCx layer 2/3. n, number of profiles analyzed; t and mem density, total and membrane-associated gold particle density, respectively. Column on the right (WT vs KO) reports comparisons between WT and KO (Mann-Whitney tests). Areas of all compartments sampled for quantitative analysis were comparable between WT and KO. With the exception of pyramidal nuclei (background), t densities and mem densities of WT were significantly higher than those of KO. °°, § comparison (Mann-Whitney tests) between densities of background and of membrane-associated gold particles in WT (°°) and KO (§) mice. In prox den, dist den, AxT, and AsP of WT mice, densities of membrane-associated gold particles were significantly higher than that of pyramidal nuclei (P=0.004, P=0.022, and P=0.024, and P<0.0001, respectively), whereas in prox den, dist den, AxT, and AsP of SSTR4 KO mouse they were significantly lower than background or comparable to background (P=0.044, P=0.002, P=0.004, and P=0.071, respectively) showing that immunogold-labelling was virtually absent at the membranes of SSTR4 KO mice.

Supplementary Table 4a

P values for Figure 2

	Soma (Fig. 2a)			(Fig. 2d)
**PV interneuron stimulation**	Active ROIs	Events/min/ROI	Amp. (dF/F0)	Cumulative
10(I) vs Bsl	0.653	0.875	0.750	0.999
10(II) vs Bsl	0.653	0.855	0.156	0.999
10(II) vs 10(II)	1,000	0.406	0.218	0.971
30(I) vs Bsl	0.312	0.203	0.326	0.502
30(II) vs Bsl	0.653	0.406	0.312	0.971
30(II) vs 30(I)	0.179	0.265	0.382	0.999
Test	Wilcoxon signed rank	Wilcoxon signed rank	Mann-Whitney	Kolmogorov-Smirnov
	**Proximal processes (Fig. 2b)**			**(Fig. 2d)**
	Active ROIs	Events/min/ROI	Amp. (dF/F0)	Cumulative
10(I) vs Bsl	0.789	1.000	0.769	1.000
10(II) vs Bsl	0.858	0.843	0.092	1.000
10(II) vs 10(II)	0.939	0.875	0.389	1.000
30(I) vs Bsl	0.010	0.017	0.005	P<0.0001
30(II) vs Bsl	0.304	0.528	0.032	0.951
30(II) vs 30(I)	0.025	0.037	0.909	0.029
Test	Wilcoxon signed rank	Wilcoxon signed rank	Mann-Whitney	Kolmogorov-Smirnov
	**Microdomains (Fig. 2c)**			**(Fig. 2d)**
	Active ROIs	Events/min/astrocyte	Amp. (dF/F0)	Cumulative
10(I) vs Bsl	0.214	0.557	0.959	0.997
10(II) vs Bsl	0.268	0.245	0.276	0.189
10(II) vs 10(II)	0.684	0.310	0.370	0.290
30(I) vs Bsl	0.009	0.014	0.888	P<0.0001
30(II) vs Bsl	0.629	0.921	0.742	P<0.0001
30(II) vs 30(I)	0.021	0.018	0.863	0.018
Test	Wilcoxon signed rank	Wilcoxon signed rank	Mann-Whitney	Kolmogorov-Smirnov
	**Soma (Fig. 2a)**			**(Fig. 2d)**
**SST interneuron stimulation**	Active ROIs	Events/min/ROI	Amp. (dF/F0)	Cumulative
10(I) vs Bsl	1.000	0.833	0.095	1.000
10(II) vs Bsl	0.696	0.476	0.285	0.853
10(II) vs 10(II)	0.696	0.718	0.891	1.000
30(I) vs Bsl	0.250	0.671	0.285	0.987
30(II) vs Bsl	0.125	0.308	0.095	0.850
30(II) vs 30(I)	0.753	0.945	0.662	1.000
Test	Wilcoxon signed rank	Wilcoxon signed rank	Mann-Whitney	Kolmogorov-Smirnov
	**Proximal processes (Fig. 2b)**			**(Fig. 2d)**
	Active ROIs	Events/min/ROI	Amp. (dF/F0)	Cumulative
10(I) vs Bsl	0.012	0.003	0.022	0.336
10(II) vs Bsl	0.002	0.006	P<0.0001	0.023
10(II) vs 10(II)	0.762	0.020	0.118	0.908
30(I) vs Bsl	0.007	0.003	P<0.0001	0.010
30(II) vs Bsl	0.002	P<0.0001	P<0.0001	P<0.0001
30(II) vs 30(I)	0.286	0.040	0.285	0.043
Test	Wilcoxon signed rank	Wilcoxon signed rank	Mann-Whitney	Kolmogorov-Smirnov
	**Microdomains (Fig. 2c)**			**(Fig. 2d)**
	Active ROIs	Events/min/astrocyte	Amp. (dF/F0)	Cumulative
10(I) vs Bsl	0.022	P<0.0001	0.618	P<0.0001
10(II) vs Bsl	P<0.0001	P<0.0001	0.539	P<0.0001
10(II) vs 10(II)	0.030	P<0.0001	0.789	P<0.0001
30(I) vs Bsl	P<0.0001	P<0.0001	0.254	P<0.0001
30(II) vs Bsl	P<0.0001	P<0.0001	0.079	P<0.0001
30(II) vs 30(I)	0.049	0.038	0.557	0.050
Test	Wilcoxon signed rank	Wilcoxon signed rank	Mann-Whitney	Kolmogorov-Smirnov
	**Soma (Fig. 2e)**			**(Fig. 2f)**
**OGB1 PV**	Active ROIs	Events/min/ROI	Amp. (dF/F0)	Cumulative
30(I) vs Bsl	0.004	P<0.0001	0.049	P<0.0001
30(II) vs Bsl	0.070	0.025	0.05	0.034
30(II) vs 30(I)	0.300	0.104	0.25	0.259
Test	Wilcoxon signed rank	Wilcoxon signed rank	Mann-Whitney	Kolmogorov-Smirnov
**OGB1 SST**	Active ROIs	Events/min/ROI	Amp. (dF/F0)	Cumulative
30(I) vs Bsl	0.010	P<0.0001	0.018	P<0.0001
30(II) vs Bsl	0.010	P<0.0001	0.042	P<0.0001
30(II) vs 30(I)	0.200	0.061	0.443	0.030
Test	Wilcoxon signed rank	Wilcoxon signed rank	Mann-Whitney	Kolmogorov-Smirnov

Supplementary Table 4b

P values for Figure 3

SST interneuron stimulation	Soma (Fig 3b)		
	Active ROIs	Events/min/ROI	Amp. (dF/F0)
10(I) vs Bsl	0.25	0.25	0.173
10(II) vs Bsl	0.125	0.125	0.125
10(II) vs 10(II)	0.773	0.625	0.229
30(I) vs Bsl	0.031	0.031	0.063
30(II) vs Bsl	0.008	0.008	0.001
30(II) vs 30(I)	0.5	0.188	0.609
	Wilcoxon Signed Rank test	Wilcoxon Signed Rank test	Mann-Whitney
	Proximal processes (Fig 3c)		
	Active ROIs	Events/min/ROI	Amp. (dF/F0)
10(I) vs Bsl	0.067	P<0.0001	0.072
10(II) vs Bsl	0.05	P<0.0001	0.034
10(II) vs 10(II)	0.537	0.56	0.532
30(I) vs Bsl	0.002	P<0.0001	0.017
30(II) vs Bsl	0.007	P<0.0001	0.012
30(II) vs 30(I)	0.594	0.755	0.164
	Paired Sample t Test	Wilcoxon Signed Rank test	Mann-Whitney
	Microdomains (Fig.3d)		
	Active ROIs	Events/min/astrocyte	Amp. (dF/F0)
10(I) vs Bsl	0.027	0.014	0.056
10(II) vs Bsl	0.025	0.005	0.285
10(II) vs 10(II)	0.388	0.045	0.32
30(I) vs Bsl	0.008	0.007	0.875
30(II) vs Bsl	0.002	0.003	0.076
30(II) vs 30(I)	0.003	0.049	0.054
	Paired Sample t Test	Paired Sample t Test	Mann-Whitney

Supplementary Table 4c

P values for Figure 4

	Soma (Fig. 4c)			(Fig. 4d)
	P Values			
**PV interneuron stimulation**	**Active ROIs**	**Events/min/ROI**	**Amp. (dF/F0)**	**Cumulative**
30(I) vs Bsl	0.042	0.002	0.005	0.039
30(II) vs Bsl	0.057	0.395	0.002	0.932
30(II) vs 30(I)	0.059	0.023	0.553	0.049
SCH vs Bsl	0.883	0.085	0.170	
Test	Paired Sample t Test	Wilcoxon signed rank	Mann-Whitney	Kolmogorov-Smirnov
**SST interneuron stimulation**	**Active ROIs**	**Events/min/ROI**	**Amp. (dF/F0)**	**Cumulative**
30(I) vs Bsl	0.010	P<0.0001	0.003	P<0.0001
30(II) vs Bsl	P<0.0001	P<0.0001	0.002	P<0.0001
30(II) vs 30(I)	0.275	0.087	0.645	0.031
SCH vs Bsl	0.500	0.499	0.116	
Test	Paired Sample t Test	Wilcoxon signed rank	Mann-Whitney	Kolmogorov-Smirnov

Supplementary Table 4d

P values for Figure 6

	Soma (Fig. 6b)		
**SST**	Active ROIs	Events/min/ROI	
SST vs Bsl	0.600	1,000	
Test	Wilcoxon signed rank	Wilcoxon signed rank	
	Proximal processes (Fig. 6b)		
	Active ROIs	Events/min/ROI	
SST vs Bsl	0.199	0.050	
Test	Wilcoxon signed rank	Wilcoxon signed rank	
	Microdomains (Fig. 6b)		
	Active ROIs	Events/min/ROI	
SST vs Bsl	0.330	0.001	
Test	Wilcoxon signed rank	Wilcoxon signed rank	
	Soma (Fig. 6c)		
**SST+ antagonists**	Active ROIs	Events/min/ROI	
SST vs Bsl	0.5	1	
Test	Wilcoxon signed rank	Wilcoxon signed rank	
	Proximal processes (Fig. 6c)		
	Active ROIs	Events/min/ROI	
SST vs Bsl	0.063	0.018	
Test	Wilcoxon signed rank	Wilcoxon signed rank	
	Microdomains (Fig. 6c)		
	Active ROIs	Events/min/ROI	
SST vs Bsl	0.073	P<0.0001	
Test	Paired t test	Wilcoxon signed rank	
	Soma (Fig. 6e)		
**CYN 154806**	Active ROIs	Events/min/ROI	Amp. (dF/F0)
10(I) vs Bsl	0.500	0.765	0.237
10(II) vs Bsl	0.875	0.531	0.200
10(II) vs 10(II)	1,000	1.000	1.000
30(I) vs Bsl	0.0008	P<0.0001	P<0.0001
30(II) vs Bsl	0.003	P<0.0001	P<0.0001
30(II) vs 30(I)	0.565	0.224	0.467
Test	Wilcoxon signed rank	Wilcoxon signed rank	Mann-Whitney
	Proximal processes (Fig. 6e)		
	Active ROIs	Events/min/ROI	Amp. (dF/F0)
10(I) vs Bsl	0.523	0.425	0.428
10(II) vs Bsl	0.771	0.212	0.857
10(II) vs 10(II)	0.557	0.669	0.999
30(I) vs Bsl	0.001	P<0.0001	P<0.0001
30(II) vs Bsl	0.005	P<0.0001	P<0.0001
30(II) vs 30(I)	0.184	0.0236	0.154
Test	Wilcoxon signed rank	Wilcoxon signed rank	Mann-Whitney
	Microdomains (Fig. 6e)		
	Active ROIs	Events/min/astrocyte	Amp. (dF/F0)
10(I) vs Bsl	0.931	0.089	1,000
10(II) vs Bsl	0.621	0.083	0.612
10(II) vs 10(II)	0.730	0.556	1.000
30(I) vs Bsl	0.010	0.005	1.000
30(II) vs Bsl	0.042	0.275	1.000
30(II) vs 30(I)	0.007	0.003	0.800
Test	Wilcoxon signed rank	Wilcoxon signed rank	Mann-Whitney

Supplementary Table 4e

P values for Figure 7

	Soma (Fig. 7a)			Fig. 7d
	Active ROIs	Events/min/ROI	Amp. (dF/F0)	Cum. Prob Events/min/ROI (origin)
10(I) vs Bsl	1	1	n/a	1.0
10(II) vs Bsl	0.5	0.5	n/a	0.873
10(II) vs 10(II)	0.5	0.5	n/a	0.873
30(I) vs Bsl	0.5	0.5	n/a	0.873
30(II) vs Bsl	0.125	0.033	n/a	0.079
30(II) vs 30(I)	0.5	0.125	n/a	0.873
	Wilcoxon signed ranks test	Wilcoxon signed ranks test	Mann-Whitney	Kolmogorov Smirnov
	Proximal processes (Fig 7b)			Fig. 7d
	Active ROIs	Events/min/ROI	Amp. (dF/F0)	Cum. Prob Events/min/ROI (clampfit)
10(I) vs Bsl	0.625	0.919	0.978	0.694
10(II) vs Bsl	1	0.748	0.86	0.772
10(II) vs 10(II)	1	0.776	0.376	0.589
30(I) vs Bsl	0.156	0.22	0.044	0.693
30(II) vs Bsl	0.031	0.266	0.521	0.694
30(II) vs 30(I)	1	0.735	0.524	0.694
	Wilcoxon signed ranks test	Wilcoxon signed ranks test	Mann-Whitney	Kolmogorov Smirnov
	Microdomains (Fig.7c)			Fig. 7d
	Active ROIs	Events/min/astrocyte	Amp. (dF/F0)	Cum. Prob Events/min/ROI (clampfit)
10(I) vs Bsl	0.492	0.836	0.441	0.671
10(II) vs Bsl	0.303	0.313	0.199	0.556
10(II) vs 10(II)	0.71	0.539	0.553	0.559
30(I) vs Bsl	0.039	0.0008	0.63	0.049
30(II) vs Bsl	0.01	0.0008	0.553	0.003
30(II) vs 30(I)	0.873	0.086	0.132	0.957
	Paired Sample t Test	Wilcoxon signed ranks test	Mann-Whitney	Kolmogorov Smirnov

Supplementary Table 4f

P values for Figure 8

PV Stim.		SST stim.			
	plus somatostatin			plus SSTRs blocker	
**30**	**30**	**10**	**30**	**10**	**30**
0.006	0.298	P<0.0001	P<0.0001	0.093	0.029
One sample Wilcoxon signed rank test		One sample Wilcoxon signed rank test		One sample Wilcoxon signed rank test	

Supplementary Table 4g

P values for Supplementary Figure 3

	p values (ANOVA)						
	PV interneuron stimulation				SST interneuron stimulation		
	friedman p		n=10, 2 mice		friedman p		n=7, 2 mice
	p<0.0001				0.0022		
	Dunn's Multiple Comparison Test				Dunn's Multiple Comparison Test		
	Stim n	vs Stim n	**p value**		Stim n	vs Stim n	**p value**
Stim 0, spontaneous	0	1	p<0.0001		0	1	0.0011
Stim 1-10, blue light	0	2	0.0001		0	2	0.0033
	0	3	p<0.0001		0	3	0.0073
	0	4	0.0002		0	4	0.0048
	0	5	0.0001		0	5	0.0057
	0	6	0.0003		0	6	0.0063
	0	7	0.0003		0	7	0.0115
	0	8	0.0003		0	8	0.0075
	0	9	0.0005		0	9	0.0073
	0	10	0.0005		0	10	0.0079
	1	2	1		1	2	1
	1	3	1		1	3	1
	1	4	1		1	4	1
	1	5	1		1	5	1
	1	6	1		1	6	1
	1	7	1		1	7	1
	1	8	1		1	8	1
	1	9	1		1	9	1
	1	10	1		1	10	1
	2	3	1		2	3	1
	2	4	1		2	4	1
	2	5	1		2	5	1
	2	6	1		2	6	1
	2	7	1		2	7	1
	2	8	1		2	8	1
	2	9	1		2	9	1
	2	10	1		2	10	1
	3	4	1		3	4	1
	3	5	1		3	5	1
	3	6	1		3	6	1
	3	7	1		3	7	1
	3	8	1		3	8	1
	3	9	1		3	9	1
	3	10	1		3	10	1
	4	5	1		4	5	1
	4	6	1		4	6	1
	4	7	1		4	7	1
	4	8	1		4	8	1
	4	9	1		4	9	1
	4	10	1		4	10	1
	5	6	1		5	6	1
	5	7	1		5	7	1
	5	8	1		5	8	1
	5	9	1		5	9	1
	5	10	1		5	10	1
	6	7	1		6	7	1
	6	8	1		6	8	1
	6	9	1		6	9	1
	6	10	1		6	10	1
	7	8	1		7	8	1
	7	9	1		7	9	1
	7	10	1		7	10	1
	8	9	1		8	9	1
	8	10	1		8	10	1
	9	10	1		9	10	1

Supplementary Table 4h

P values for Supplementary Figure 4

	Soma (Fig. 4b)		Soma (Fig. 4d)
**PV 10s**	n. of Ca2+ events	**SST 10s**	n. of Ca2+ events
10	1,000	10	1,000
20	1,000	20	0.861
30	0.154	30	0.428
40	0.209	40	1,000
50	1,000	50	0.001
60	1,000	60	0.428
Test	Wilcoxon signed rank	Test	Wilcoxon signed rank
	Proximal processes (Fig. 4b)		Proximal processes (Fig. 4d)
	n. of Ca2+ events		n. of Ca2+ events
10	0.874	10	0.821
20	0.382	20	0.821
30	0.122	30	0.821
40	0.431	40	0.076
50	1,000	50	0.001
60	1,000	60	0.481
Test	Wilcoxon signed rank	Test	Wilcoxon signed rank
	Microdomains (Fig. 4b)		Microdomains (Fig. 4d)
	n. of Ca2+ events		n. of Ca2+ events
10	0.924	10	0.691
20	0.192	20	0.019
30	0.076	30	0.004
40	0.655	40	0.017
50	0.924	50	0.0005
60	0.997	60	0.0008
Test	Wilcoxon signed rank	Test	Wilcoxon signed rank
	Soma (Fig. 4b)		Soma (Fig. 4d)
**PV 30s**	n. of Ca2+ events	**SST 30s**	n. of Ca2+ events
10	1,000	10	1,000
20	1,000	20	0.735
30	1,000	30	0.731
40	0.207	40	1,000
50	0.002	50	0.002
60	0.021	60	0.101
Test	Wilcoxon signed rank	Test	Wilcoxon signed rank
	Proximal processes (Fig. 4b)		Proximal processes (Fig. 4d)
	n. of Ca2+ events		n. of Ca2+ events
10	0.709	10	0.799
20	0.103	20	0.779
30	0.103	30	0.748
40	0.084	40	0.331
50	0.002	50	0.004
60	0.003	60	0.003
Test	Wilcoxon signed rank	Test	Wilcoxon signed rank
	Microdomains (Fig. 4b)		Microdomains (Fig. 4d)
	n. of Ca2+ events		n. of Ca2+ events
10	0.922	10	0.369
20	0.097	20	0.025
30	0.075	30	0.037
40	0.010	40	0.0002
50	0.050	50	p<0.0001
60	0.050	60	p<0.0001
Test	Wilcoxon signed rank	Test	Wilcoxon signed rank

Supplementary Table 4i

P values for Supplementary Figure 7

	Soma (Fig. 7a)			(Fig. 7d)
**PV interneuron stimulation**	Active ROIs	Events/min/ROI	Amp. (dF/F0)	Cumulative
10(I) vs Bsl	0.998	0.750	0.138	1.000
10(II) vs Bsl	0.998	0.812	0.148	1.000
10(II) vs 10(II)	1,000	0.906	0.333	1,000
30(I)vs Bsl	0.218	0.125	0.904	0.537
30(II) vs Bsl	0.289	0.250	0.785	0.978
30(II) vs 30(I)	0.269	0.441	0.792	0.999
SCH vs bas	0.958	0.682	0.333	/
Test	Wilcoxon signed rank	Wilcoxon signed rank	Mann-Whitney	Kolmogorov-Smirnov
	**Proximal processes (Fig. 7b)**			**(Fig. 7d)**
	Active ROIs	Events/min/ROI	Amp. (dF/F0)	Cumulative
10(I) vs Bsl	1,000	0.957	0.859	0.999
10(II) vs Bsl	0.958	0.632	0.395	0.999
10(II) vs 10(II)	0.973	0.381	0.700	0.999
30(I)vs Bsl	0.050	0.002	0.002	0.049
30(II) vs Bsl	0.593	0.027	0.040	0.778
30(II) vs 30(I)	0.035	0.025	0.237	0.033
SCH vs bas	0.921	0.881	0.200	/
Test	Wilcoxon signed rank	Wilcoxon signed rank	Mann-Whitney	Kolmogorov-Smirnov
	**Microdomains (Fig. 7c)**			**(Fig. 7d)**
	Active ROIs	Events/min/ROI	Amp. (dF/F0)	Cumulative
10(I) vs Bsl	0.624	0.741	0.837	0.999
10(II) vs Bsl	0.945	0.771	1.000	1.000
10(II) vs 10(II)	0.312	0.975	0.962	1.000
30(I)vs Bsl	0.007	p<0.0001	0.918	p<0.0001
30(II) vs Bsl	0.150	0.971	0.694	0.102
30(II) vs 30(I)	0.023	p<0.0001	0.480	0.004
SCH vs bas	0.555	0.286	0.322	/
Test	Wilcoxon signed rank	Wilcoxon signed rank	Mann-Whitney	Kolmogorov-Smirnov
	**Soma (Fig. 7a)**			**(Fig. 7d)**
**SST interneuron stimulation**	Active ROIs	Events/min/ROI	Amp. (dF/F0)	Cumulative
10(I) vs Bsl	0.179	0.138	0.546	0.753
10(II) vs Bsl	0.065	0.010	0.030	0.188
10(II) vs 10(II)	0.774	0.130	0.350	0.329
30(I) vs Bsl	0.003	p<0.0001	0.002	0.058
30(II) vs Bsl	0.001	0.008	0.002	0.099
30(II) vs 30(I)	0.932	0.342	0.707	0.525
SCH vs bas	0.922	0.944	1,000	/
Test	Wilcoxon signed rank	Wilcoxon signed rank	Mann-Witney	Kolmogorov-Smirnov
	**Proximal processes (Fig. 7b)**			**(Fig. 7d)**
	Active ROIs	Events/min/ROI	Amp. (dF/F0)	Cumulative
10(I) vs Bsl	0.019	0.012	0.623	0.092
10(II) vs Bsl	0.007	0.023	p<0.0001	0.001
10(II) vs 10(II)	0.422	0.357	0.001	0.989
30(I) vs Bsl	0.013	p<0.0001	p<0.0001	p<0.0001
30(II) vs Bsl	0.013	p<0.0001	p<0.0001	p<0.0001
30(II) vs 30(I)	0.855	0.050	0.274	0.006
SCH vs bas	0.491	0.910	0.515	/
Test	Wilcoxon signed rank	Wilcoxon signed rank	Mann-Witney	Kolmogorov-Smirnov
	**Microdomains (Fig. 7c)**			**(Fig. 7d)**
	Active ROIs	Events/min/astrocyte	Amp. (dF/F0)	Cumulative
10(I) vs Bsl	0.025	p<0.0001	0.130	p<0.0001
10(II) vs Bsl	p<0.0001	p<0.0001	0.064	p<0.0001
10(II) vs 10(II)	p<0.0001	0.002	0.796	0.010
30(I) vs Bsl	p<0.0001	p<0.0001	0.140	p<0.0001
30(II) vs Bsl	p<0.0001	p<0.0001	0.176	p<0.0001
30(II) vs 30(I)	0.001	0.002	0.198	0.018
SCH vs bas	0.550	0.457	0.921	/
Test	Wilcoxon signed rank	Wilcoxon signed rank	Mann-Witney	Kolmogorov-Smirnov

Supplementary Table 4j

P values for Supplementary Figure 9

	Soma (Fig. 9e)			(Fig.9f)
	P Values			
**PV interneuron stimulation**	Active ROIs	Events/min/ROI	Amp. (dF/F0)	Cumulative
30(I) vs Bsl	0.005	p<0.0001	0.002	0.001
30(II) vs Bsl	0.010	0.010	0.005	0.007
30(II) vs 30(I)	0.062	0.334	0.620	0.450
SCH vs Bsl	0.125	0.135	0.143	
Test	Paired Sample t Test	Wilcoxon signed rank	Mann-Whitney	Kolmogorov-Smirnov
**SST interneuron stimulation**	Active ROIs	Events/min/soma	Amp. (dF/F0)	Cumulative
30(I) vs Bsl	0.0004	p<0.0001	0.008	p<0.0001
30(II) vs Bsl	0.0001	p<0.0001	0.006	p<0.0001
30(II) vs 30(I)	0.329	0.096	0.389	0.009
SCH vs Bsl	0.354	0.625	0.772	
Test	Paired Sample t Test	Wilcoxon signed rank	Mann-Whitney	Kolmogorov-Smirnov

Supplementary Table 4k

P values for Supplementary Figure 10

		Soma (Suppl. Fig 10b)		
		Active ROIs	Events/min/ROI	Amp. (dF/F0)
PV interneuron stimulation	30(I) vs Bsl	0.25	0.125	n/a
		Wilcoxon signed rank	Wilcoxon signed rank	
SST interneuron stimulation	30(I) vs Bsl	0.61	1	n/a
		Paired t test	Paired t test	
		Proximal processes (Suppl. Fig 10c)		
		Active ROIs	Events/min/ROI	Amp. (dF/F0)
PV interneuron stimulation	30(I) vs Bsl	0.049	0.016	0.049
		Paired t test	Wilcoxon signed rank	Mann-Whitney
SST interneuron stimulation	30(I) vs Bsl	0.012	p<0.0001	p<0.0001
		Paired t test	Wilcoxon signed rank	Mann-Whitney
		Microdomains (Suppl. Fig 10d)		
		Active ROIs	Events/min/astrocyte	Amp. (dF/F0)
PV interneuron stimulation	30(I) vs Bsl	0.023	0.004	0.742
		Paired t test	Paired t test	Mann-Whitney
SST interneuron stimulation	30(I) vs Bsl	0.0185	0.007	0.507
		Paired t test	Paired t test	Unpaired t test

Supplementary Table 4l

P values for Supplementary Figure 11

SST interneuron stimulation		Soma (Suppl. Fig 11a)			Fig. 11d
Interval 30(I)-30(II)		**Active ROIs**	**Events/min/ROI**	**Amp. (dF/F0)**	**Cum. Prob Events/min/ROI**
10 min	30(I) vs Bsl	1	0.5	0.195	1.0
	30(II) vs Bsl	0.125	0.125	0.020	0.212
	30(II) vs 30(I)	0.25	0.25	0.095	0.575
20 min	30(I)	0.25	0.125	0.081	0.66
	30(II); 20 min	0.688	0.625	0.347	0.98
	30(II) vs 30(I)	1	0.625	0.295	0.98
		Wilcoxon signed ranks test	Wilcoxon signed ranks test	Mann-Whitney	Kolmogorov Smirnov
		**Proximal processes (Suppl. Fig 11b)**			**Fig. 11d**
Interval 30(I)-30(II)		**Active ROIs**	**Events/min/ROI**	**Amp. (dF/F0)**	**Cum. Prob Events/min/ROI**
10 min	30(I) vs Bsl	0.045	0.003	0.003	0.009
	30(II) vs Bsl	0.044	0.0003	0.001	0.002
	30(II) vs 30(I)	0.17	0.868	0.45	1
20 min	30(I)	0.007	0.003	0.002	0.031
	30(II); 20 min	0.006	0.015	0.002	0.022
	30(II) vs 30(I)	0.685	0.661	0.939	1.000
		Paired t test	Paired t test (10min)	Unpaired t test	Kolmogorov Smirnov
			Wilcoxon signed ranks test (20 min)		
		**Microdomains (Suppl. Fig 11c)**			**Fig. 11d**
Interval 30(I)-30(II)		**Active ROIs**	**Events/min/astrocyte**	**Amp. (dF/F0)**	**Cum. Prob Events/min/ROI**
10 min	30(I) vs Bsl	0.016	0.016	0.228	p<0.0001
	30(II) vs Bsl	0.016	0.016	0.148	p<0.0001
	30(II) vs 30(I)	0.031	0.869	0.588	0.052
20 min	30(I)	0.016	0.008	0.926	p<0.0001
	30(II); 20 min	0.008	0.016	0.579	p<0.0001
	30(II) vs 30(I)	0.688	0.688	0.456	0.809
		Wilcoxon signed ranks test	Wilcoxon signed ranks test	Unpaired t test	Kolmogorov Smirnov

Supplementary Table 4m

P values for Supplementary Figure 12

	Soma (Fig. 12c)		
**In vivo**	Active ROIs	Events/min/ROI	Amp. (dF/F0)
fist half vs second half	0.998	0.999	0.333
Test	Wilcoxon signed rank	Wilcoxon signed rank	Mann-Whitney
	Proximal processes (Fig. 12c)		
	Active ROIs	Events/min/ROI	Amp. (dF/F0)
fist half vs second half	0.876	0.615	0.309
Test	Wilcoxon signed rank	Wilcoxon signed rank	Mann-Whitney
	Microdomains (Fig. 12c)		
	Active ROIs	Events/min/ROI	Amp. (dF/F0)
fist half vs second half	0.707	0.989	0.321
Test	Wilcoxon signed rank	Wilcoxon signed rank	Mann-Whitney
	Soma (Fig. 12f)		
**In slice**	Active ROIs	Events/min/ROI	Amp. (dF/F0)
fist half vs second half	0.953	0.794	0.400
Test	Wilcoxon signed rank	Wilcoxon signed rank	Mann-Whitney
	Proximal processes (Fig. 12f)		
	Active ROIs	Events/min/ROI	Amp. (dF/F0)
fist half vs second half	0.938	0.657	0.979
Test	Wilcoxon signed rank	Wilcoxon signed rank	Mann-Whitney
	Microdomains (Fig. 12f)		
	Active ROIs	Events/min/ROI	Amp. (dF/F0)
fist half vs second half	0.948	0.515	0.547
Test	Wilcoxon signed rank	Wilcoxon signed rank	Mann-Whitney

Supplementary Table 4n		
**P values for Supplementary Figure 13**		
		**Suppl. Fig 13 f,l**
		**P Values**
		**IPSCs**
**PV interneuron stimulation**	10(II) vs 10(I)	0.77
	30(II) vs 30(I)	0.55
		Paired t test
**SST interneuron stimulation**	10(II) vs 10(I)	0.76
	30(II) vs 30(I)	0.34
		Paired t test
		**Suppl. Fig 13 c,i**
		**P Values**
		**Firing (Hz)**
**PV interneuron stimulation**	10(II) vs 10(I)	0.165
	30(II) vs 30(I)	0.318
		Paired t test
**SST interneuron stimulation**	10(II) vs 10(I)	0.185
	30(II) vs 30(II)	0.829
		Paired t test
