# Supplementary material for: Interneuron-specific signaling evokes distinctive somatostatin-mediated responses in adult cortical astrocytes
Source: Nat Commun. 2018 Jan 8;9:82. doi: 10.1038/s41467-017-02642-6 (PMC5758790; doi:10.1038/s41467-017-02642-6)
Supplement: Supplementary file 3 — Description of Additional Supplementary Files [file 41467_2017_2642_MOESM3_ESM.pdf]

File Name: Supplementary Movie 1
Description: To facilitate comparisons, in Movie 1 we combined three movies, each of 160 seconds, illustrating the Ca^{2+} signal dynamics at basal conditions (bsl), after 10 (Stim 10) and 30 (Stim 30) pulse activation of PV interneurons in a GCaMP6f-astrocyte *in vivo* (the same astrocyte of Figure 1B) from layer 2/3 SSCx of a ChR2-PV-GCaMP6f mouse. Frame rate: 15 frames per second. Acquisition time frame, 1 Hz. Scale bar, 10 µm.

File Name: Supplementary Movie 2
Description: Combined Movie 2 (each movie, 160 seconds) illustrates the Ca^{2+} signal dynamics at basal conditions (bsl), after 10 (Stim 10) and 30 (Stim 30) pulse activation of SST interneurons in a GCaMP6f-astrocyte *in vivo* (the same astrocyte of Figure 1E) from layer 2/3 SSCx of a ChR2-SST-GCaMP6f mouse. Frame rate: 15 frames per second. Acquisition time frame, 1 Hz. Scale bar, 10 µm.

File Name: Supplementary Movie 3
Description: The combined Movie 3 (each movie, 107 seconds) illustrates the Ca^{2+} signal dynamics in layer 2/3 SSCx GCaMP6f-astrocytes in brain slice preparations from a ChR2-SST-GCaMP6f mouse at basal conditions (bsl), after 10 (Stim 10) and 30 (Stim 30) pulse activation of SST interneurons performed in the presence of the SSTR antagonist CYN154806. Frame rate: 30 frames per second. Acquisition time frame, 3.65 Hz. Scale bar, 10 µm.
